# Usher syndrome type 1-associated gene, *pcdh15b*, is required for photoreceptor structural integrity in zebrafish

**DOI:** 10.1242/dmm.048965

**Published:** 2021-12-07

**Authors:** Amanda Miles, Clarke Blair, Andrew Emili, Vincent Tropepe

**Affiliations:** 1 Department of Cell and Systems Biology, University of Toronto, Toronto, ON M5S 3G5, Canada; 2 Center for Network Systems Biology, Boston University, Boston, MA 02118, USA

**Keywords:** Calyceal processes, Outer segment, Retina, Usher syndrome, Zebrafish, Photoreceptor

## Abstract

Blindness associated with Usher syndrome type 1 (USH1) is typically characterized as rod photoreceptor degeneration, followed by secondary loss of cones. The mechanisms leading to blindness are unknown because most genetic mouse models only recapitulate auditory defects. We generated zebrafish mutants for one of the USH1 genes, *protocadherin-15b* (*pcdh15b*), a putative cell adhesion molecule. Zebrafish Pcdh15 is expressed exclusively in photoreceptors within calyceal processes (CPs), at the base of the outer segment (OS) and within the synapse. In our mutants, rod and cone photoreceptor integrity is compromised, with early and progressively worsening abnormal OS disc growth and detachment, in part due to weakening CP contacts. These effects were attenuated or exacerbated by growth in dark and bright-light conditions, respectively. We also describe novel evidence for structural defects in synapses of *pcdh15b* mutant photoreceptors. Cell death does not accompany these defects at early stages, suggesting that photoreceptor structural defects, rather than overt cell loss, may underlie vision deficits. Thus, we present the first genetic animal model of a *PCDH15*-associated retinopathy that can be used to understand the aetiology of blindness in USH1.

This article has an associated First Person interview with the first author of the paper.

## Abstract (precis)

**Summary:** We present one of the first genetic animal mutants for *PCDH15* that displays a severe, early retinopathy and suggests that zebrafish could be a useful model for *PCDH15*-associated retinal phenotypes.

## INTRODUCTION

Usher syndrome (USH) is an autosomal recessive condition that results in progressive deafness and blindness. Three clinical subtypes of the disease are recognized, and, of these, USH1 is the most prevalent and severe form (Mathur and Yang, 2015). USH1 is characterized by severe to profound congenital deafness, vestibular defects and a pre-pubertal onset of retinitis pigmentosa (RP). RP is characterized by progressive primary loss of rod photoreceptors followed by secondary loss of cone photoreceptors (El-Amraoui and Petit, 2014). Mutations in six genes have been linked to USH1 so far. These include the cell adhesion molecules cadherin-23 (*CDH23*/*USH1D*) and protocadherin-15 (*PCDH15*/*USH1F*), scaffolding proteins harmonin (*USH1C*) and sans (*USH1G*), motor protein myosin VIIa (*MYO7A*/*USH1B*) and the calcium and integrin binding protein *CIB2* (*USH1J*) (Mathur and Yang, 2015).

Our understanding of the function of these USH1 genes comes primarily from studies in the ear (El-Amraoui and Petit, 2014; Mathur and Yang, 2015). These studies have shown that USH1 proteins function to link together actin-rich microvilli called stereocilia, in neurosensory hair cells of the inner ear. To bridge together adjacent stereocilia, *Pcdh15* and *Cdh23* interact via the ends of their extracellular cadherin domains to form a structural fibre known as a ‘tip link’ (Kazmierczak et al., 2007). The other USH1 proteins form an intracellular complex to help anchor the tip links to the F-actin filaments present in the stereocilia (El-Amraoui and Petit, 2014; Mathur and Yang, 2015). When stereocilia are deflected by sound, PCDH15-CDH23 are stretched, opening physically linked TMC1/2 channels, causing cellular depolarization (Maeda et al., 2014; Pan et al., 2018). The current model suggests that USH1 proteins coordinate tip-link adhesion and tension to regulate auditory mechanotransduction. However, whether USH1 protein-dependent adhesion and tension are required for the proper functioning and integrity of photoreceptors is less clear.

USH1 mouse models have aided in our understanding of structural and functional auditory defects associated with the disorder, but most models fail to display retinal defects (reviewed in El-Amraoui and Petit, 2014). Specifically, although some deficiencies in vision have been reported, no mouse mutant has shown retinal degeneration/morphology defects unless exposed to altered light conditions (i.e. enhanced light/lack of pigmentation) (Calabro et al., 2019; Haywood-Watson et al., 2006; Lentz et al., 2010; Libby and Steel, 2001; Libby et al., 2003; Peng et al., 2011; Trouillet et al., 2018; Williams et al., 2009). One possible explanation is that mice exhibit a photoreceptor structure that diverges from that of humans and other species (El-Amraoui and Petit, 2014). For example, USH1 proteins have been found to localize to F-actin-rich microvilli-like protrusive structures, called calyceal processes (CPs), that surround outer segments (OSs) of photoreceptors (Sahly et al., 2012). CPs appear to be missing from mouse photoreceptors, suggesting that a lack of overt retinopathy in mouse models might be due to species differences. This highlights the need for an accurate and reliable genetic animal model of USH1-associated retinopathy.

Recent knockdowns of *pcdh15* and *cdh23* in *Xenopus* suggest that, like in tip links, Pcdh15-Cdh23 structures ‘link’ CPs to the OS to provide the structural support necessary for proper cone and rod OS shape (Schietroma et al., 2017). Zebrafish photoreceptors have similar CPs (Hodel et al., 2014; Tarboush et al., 2012), yet zebrafish mutant models of various USH1-related genes have shown inconsistent or mild consequences to photoreceptor structure or survival (Glover et al., 2012; Hodel et al., 2014; Phillips et al., 2011; Wasfy et al., 2014). For instance, although *cdh23* is expressed in the zebrafish retina, it has not been detected in photoreceptors, nor have *cdh23* mutants shown defects in the morphology and function of photoreceptors (Glover et al., 2012). Therefore, there is a need for new animal models to decipher the precise function of USH1-related genes in photoreceptors. Additionally, most USH1 gene expression has also been described in photoreceptor synapses, suggesting that multiple roles of USH1 proteins may exist, but current analysis has been largely focused on the OS (Reiners et al., 2005).

In this study we focused on *PCDH15* (*USH1F*), which is associated with up to 20% of USH1 cases (Ouyang et al., 2005; Roux et al., 2006), to generate the first *PCDH15* genetic zebrafish model displaying a clear retinopathy. Zebrafish have two *PCDH15* paralogs: *pcdh15a* and *pcdh15b* (Seiler et al., 2005). Studies of the *pcdh15a* ‘orbiter’ mutant suggest that *pcdh15a* has a highly conserved function in stereocilia morphology and inner ear hair cell function but no retinal phenotype (Maeda et al., 2017; Seiler et al., 2005). In contrast, *pcdh15b* mRNA is expressed in photoreceptors of the zebrafish embryonic retina, but until now there has been limited evidence for a functional role in the retina (Seiler et al., 2005). We show that Pcdh15a/b is expressed in the zebrafish retina in photoreceptors at the CPs, inner segment (IS)-OS junction and synapse. Using loss-of-function *pcdh15b* mutants generated by CRISPR, we examined the effect on photoreceptors and demonstrate an early, progressively worsening defect in OS morphology and attachment, as well as synaptic disorganization. These structural defects preceded typical signs of photoreceptor cell death. We also found a strong defect in cone morphology, which suggests a distinct aetiology compared to a typical RP phenotype previously assumed to underlie the USH1 retinopathy.

## RESULTS

### Pcdh15 is expressed in photoreceptors of the zebrafish retina

**Fig. 1.**
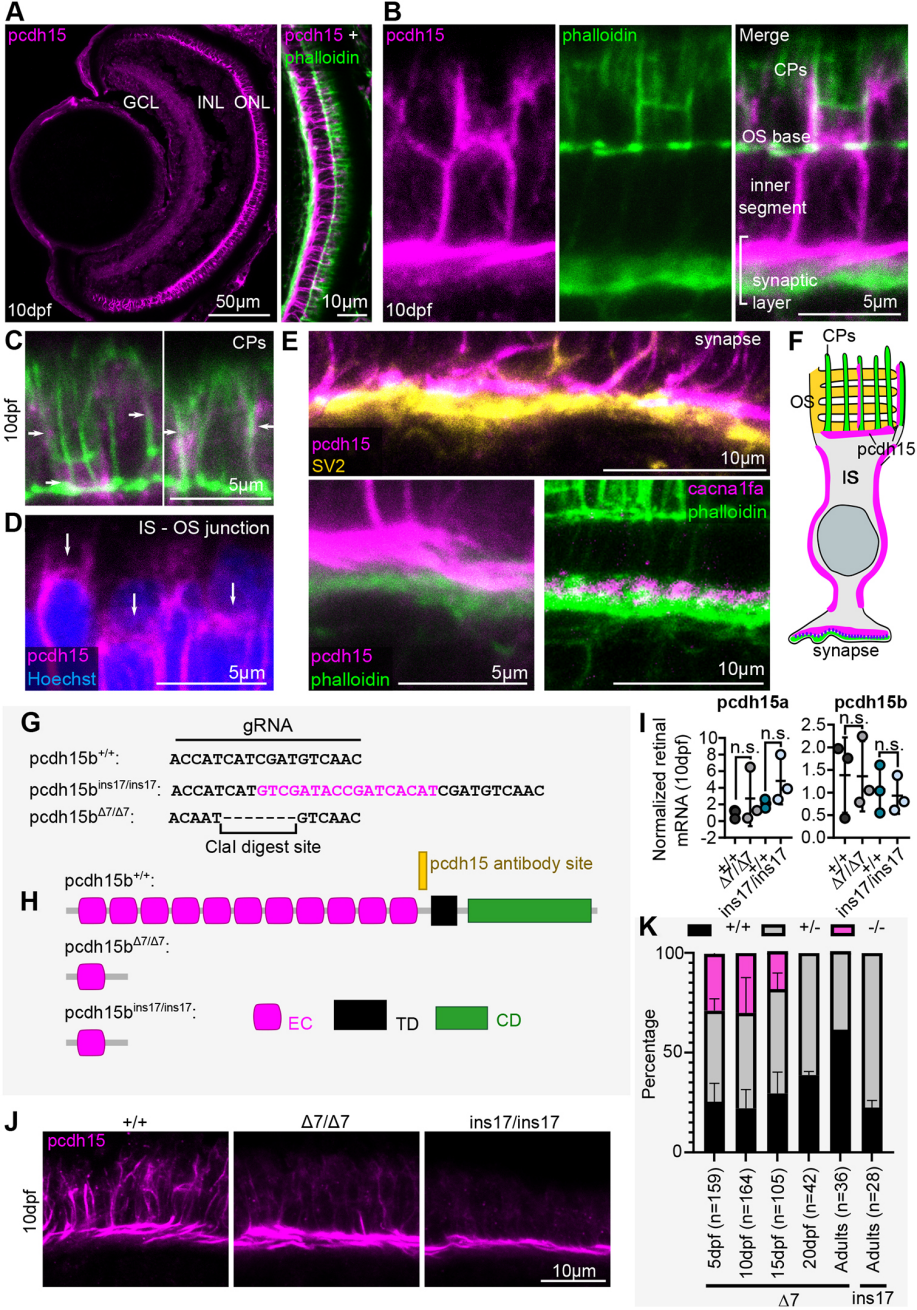
**Pcdh15 is expressed in the photoreceptors of the zebrafish retina and is reduced in CRISPR-generated *pcdh15b* mutants.** (A) Representative images of the 10 dpf retina stained for Pcdh15 (magenta). Co-label with F-actin stain, phalloidin (green; present in the CPs and synapse) is shown for the central ONL. (B) Close up of a central region in the ONL stained for Pcdh15 (magenta), phalloidin (green) and an overlap of the two. (C,D) Close up of the co-staining for Pcdh15 and phalloidin in the CPs (C) and for Pcdh15 at the IS-OS junction (D). (E) Representative images of SV2 staining (yellow) and calcium channel 1.4_v_ staining (Cacna1fa; magenta) in the photoreceptor synapse and colocalization with Pcdh15 and phalloidin (green) at 10 dpf. (F) Schematic of Pcdh15 expression in the zebrafish photoreceptor cell. Green represents phalloidin in the synapse and CPs; magenta represents Pcdh15 in the CPs, OS base, IS membrane and synapse; blue dots represent calcium channel 1.4_v_ in the synapse. (G) gRNA used to target and mutate the *pcdh15b* gene. A 17 bp insertion (magenta nucleotides) and a 7 bp deletion (black nucleotides) mutant were identified. (H) Predicted results of the mutations on the protein sequence. (I) RT-qPCR to analyse *pcdh15a* (left) and *pcdh15b* (right) mRNA expression, normalized to actin, in wild-type siblings and *pcdh15b* mutants. (J) Staining for Pcdh15 in wild-type siblings (+/+) and mutants (Δ7/Δ7 and ins17/ins17) at 10 dpf (*n*=5+ for each genotype). (K) Genotyping of clutches of heterozygous crosses up to adulthood to measure survival [numbers of fish genotyped (*n*) are indicated]. CD, variable cytoplasmic domain; CP, calyceal process; EC, extracellular cadherin domains; GCL, ganglion cell layer; INL, inner nuclear layer; IS, inner segment; ONL, outer nuclear layer; OS, outer segment; TD, transmembrane domain.

We first sought to determine the expression and localization of Pcdh15 in the zebrafish retina. Expression of all USH1 proteins, including PCDH15, in *Xenopus*, macaque and humans has largely been observed in photoreceptors, and recently described in CPs (Sahly et al., 2012). Within photoreceptors, USH1 proteins have also been described in different regions of the OS, IS and synapse (Lagziel et al., 2009; Reiners et al., 2003). Moreover, particularly in zebrafish, expression of *cdh23* and *harmonin* has been described in other retinal cells, such as amacrine cells and Müller glia (Glover et al., 2012; Phillips et al., 2011). To determine Pcdh15 expression in zebrafish, we used a commercially available anti-Pcdh15 antibody designed against the C-terminal region of the protein. This region is present in all described zebrafish Pcdh15b isoforms as well as Pcdh15a isoforms, with 83% identity to both Pcdh15a and Pcdh15b (Fig. S1A,B). Using this antibody, expression was observed exclusively in photoreceptors at 3 days post-fertilization (dpf) and 10 dpf (Fig. 1A; Fig. S1C). We co-labelled with phalloidin, which labels F-actin present in CPs, and found that although Pcdh15 does colocalize with phalloidin-labelled CPs (Fig. 1A-C,F), Pcdh15 expression is also found in basolateral regions of the photoreceptor (Fig. 1A,B,D,F). Pcdh15 was also found at the IS-OS junction and along the IS plasma membrane (Fig. 1B,D,F). Expression was also observed in the presumptive synaptic region of photoreceptors as early as 3 dpf, although it does not directly overlap with synaptic actin or SV2, a synaptic vesicle marker at 10 dpf (Fig. 1B,E; Fig. S1C). To determine whether Pcdh15 may interact with important synaptic components, we examined the localization of photoreceptor synaptic calcium channel 1.4_v_, Cacna1fa, and found that it had minimal overlap with F-actin (Fig. 1E). Use of all these markers places Pcdh15 expression immediately adjacent to synaptic components (Fig. 1E,F). Therefore, zebrafish Pcdh15 expression is present in CPs, but is also present at the OS base, IS plasma membrane and synapse of the zebrafish photoreceptor. The specificity of the antibody for Pcdh15 is supported by western blot analysis, which showed similar-sized isoforms to those previously observed in the mouse retina using a C-terminal antibody (Zallocchi et al., 2012) (Fig. S1D). The specificity for the Pcdh15b paralog is supported by an observed reduction in labelling in *pcdh15b* mutant retinas (described below). However, this antibody is expected to recognize both Pcdh15a and Pcdh15b (see Fig. S1B) and therefore this expression pattern may represent a combination of both proteins. In support of this possibility, we examined *pcdh15a* mRNA expression by reverse transcription quantitative PCR (RT-qPCR) and found that it was detected in zebrafish eyes from 3 dpf to 10 dpf at comparable levels of expression to *pcdh15b* (Fig. 1I; Fig. S1E). These data suggest that Pcdh15 proteins are expressed in different subcompartments of photoreceptors in the zebrafish retina.

### CRISPR-generated *pcdh15b* zebrafish mutants show reduced Pcdh15 staining in the retina and reduced survival

Because *pcdh15a* mutants do not show compromised retinal function, and previous studies suggest that *pcdh15b* may have a photoreceptor-specific function (Seiler et al., 2005), we aimed to mutate the *pcdh15b* zebrafish paralog by CRISPR gene-editing technology. We used a guide RNA (gRNA) for *pcdh15b*, targeting a common exon predicted to be present in all alternatively spliced products described thus far. In this case, exon 7 was targeted to ensure that most, if not all, *pcdh15b* products would be affected (Fig. 1G). We identified two mutations for *pcdh15b*, which we chose to focus on for further analysis: a 7 bp deletion mutant (referred to as Δ7) and a 17 bp insertion mutant (referred to as ins17) (Fig. 1G; Fig. S1F-H). Both mutations are predicted to severely truncate *pcdh15b* by introducing a premature stop codon in the open reading frame, removing nearly all the extracellular cadherin domain repeats, the transmembrane domain and the cytoplasmic domain (Fig. 1H). Both *pcdh15a* and *pcdh15b* mRNA can be detected in the retina, but no differences in their mRNA levels were observed from the eyes of Δ7 and ins17 mutants (Fig. 1I; Fig. S1E), although we confirmed that the mutation was present in the mRNA product by sequencing. In contrast, we found that *pcdh15b* mutants had reduced Pcdh15 protein expression by immunolabelling, namely along the CPs, cell body and IS-OS base (Fig. 1J). Using the same C-terminal antibody, we observed a reduction in overall protein levels in mutants by western blot analysis (Fig. S1D). Some of the isoforms appeared to be more affected than others, which might correlate with the residual expression we observed by immunolabelling, particularly in the synapse. This is consistent with mouse *Pcdh15* mutants that also demonstrate persistent expression of some isoforms (Alagramam et al., 2011). In addition, our immunolabelling and western blot analyses could be detecting the presence of Pcdh15a isoforms.

We observed that homozygous mutants, with either mutant allele, had significantly reduced survival to adulthood (Fig. 1K). To analyse this further, we focused on the Δ7 mutants. We genotyped multiple clutches from heterozygous crosses of Δ7 mutants at different time points during development to determine the ratios of each genotype present. We found that all Δ7 mutants die by 20 dpf (Fig. 1K). Given the comparable phenotypes observed in homozygous mutant larvae from the two separately out-crossed mutant lines, we assume that these phenotypes are specifically associated with a loss-of-function mutation in *pcdh15b*. Currently, we do not know why homozygous fish die, as we have not identified any overt morphological defects in homozygous fish and feeding behaviour does not seem to be affected. *pcdh15a* ‘orbiter’ mutants also have reduced survival (Nicolson et al., 1998), and it has been previously observed that *pcdh15a* and *pcdh15b* expression are not limited to the ear and retina, respectively (Seiler et al., 2005). Therefore, other tissues with microvilli-like protrusions, such as the brain, in which *pcdh15b* is highly expressed (Seiler et al., 2005), may be affected in these mutants.

### Hair cell defects are not detected in the inner ear and lateral line of *pcdh15b* mutants

Studies have found that *Pcdh15* predominately functions at ‘tip links’ of stereocilia in hair cells to adhere multiple stereocilia into bundled tips (Kazmierczak et al., 2007). In zebrafish, this occurs in hair cells of the inner ear as well as in sensory hair cells along the lateral line, found in structures called neuromasts (Maeda et al., 2017). A ‘splayed’ degenerative phenotype of tip ends in the ear or neuromasts is commonly observed in *pcdh15a* mutants by 5 dpf (Goodman and Zallocchi, 2017; Maeda et al., 2017; Seiler et al., 2005). Similar to *pcdh15a*, some expression of *pcdh15b* has also previously been described in the hair cells of the ear and lateral line (Seiler et al., 2005) but it is unclear whether its loss results in a phenotype. We utilized whole-mount immunostaining of the inner ear and scanning electron microscopy (SEM) of neuromasts to visualize these bundled tips. After staining and imaging for phalloidin to label F-actin-rich stereocilia in the medial crista of the inner ear, we found that *pcdh15b* mutants showed intact bundled ends, at 5 dpf and even 10 dpf, suggesting no degenerative phenotype in these cells (Fig. S2A). In addition, we found that neuromasts showed intact tip ends in our *pcdh15b* mutants, comparable to those of wild-type siblings, at 10 dpf (Fig. S2B,C). Therefore, unlike *pcdh15a* zebrafish mutants and *Pcdh15* mouse mutants, our *pcdh15b* zebrafish mutants do not appear to show abnormal hair cell morphology, although we cannot rule out subtle morphological defects. However, unlike *pcdh15a* ‘orbiter’ mutants, which exhibit variable degrees of abnormal ‘looping’ and ‘tilted’ swimming (Nicolson et al., 1998), no detectable change in swimming behaviour is observed in our *pcdh15b* mutants.

### Photoreceptor morphology in *pcdh15b* mutant retinas is abnormal despite no outer nuclear layer (ONL) thinning

The lack of retinal phenotypes observed in mouse models of *Pcdh15* and other associated USH genes has limited our understanding of the retinopathy occurring in this disorder (El-Amraoui and Petit, 2014). According to our protein expression analysis, as well as that seen in other species (Sahly et al., 2012; Schietroma et al., 2017), Pcdh15 activity and function appears specific to photoreceptors. However, because other USH1 proteins have been described in other retinal cell types, we first assessed the phenotypes of other retinal cell types in our *pcdh15b* mutants and found no overt abnormalities in ganglion cells, amacrine cells or Müller glia at 10 dpf (Fig. S3A,B).

**Fig. 2.**
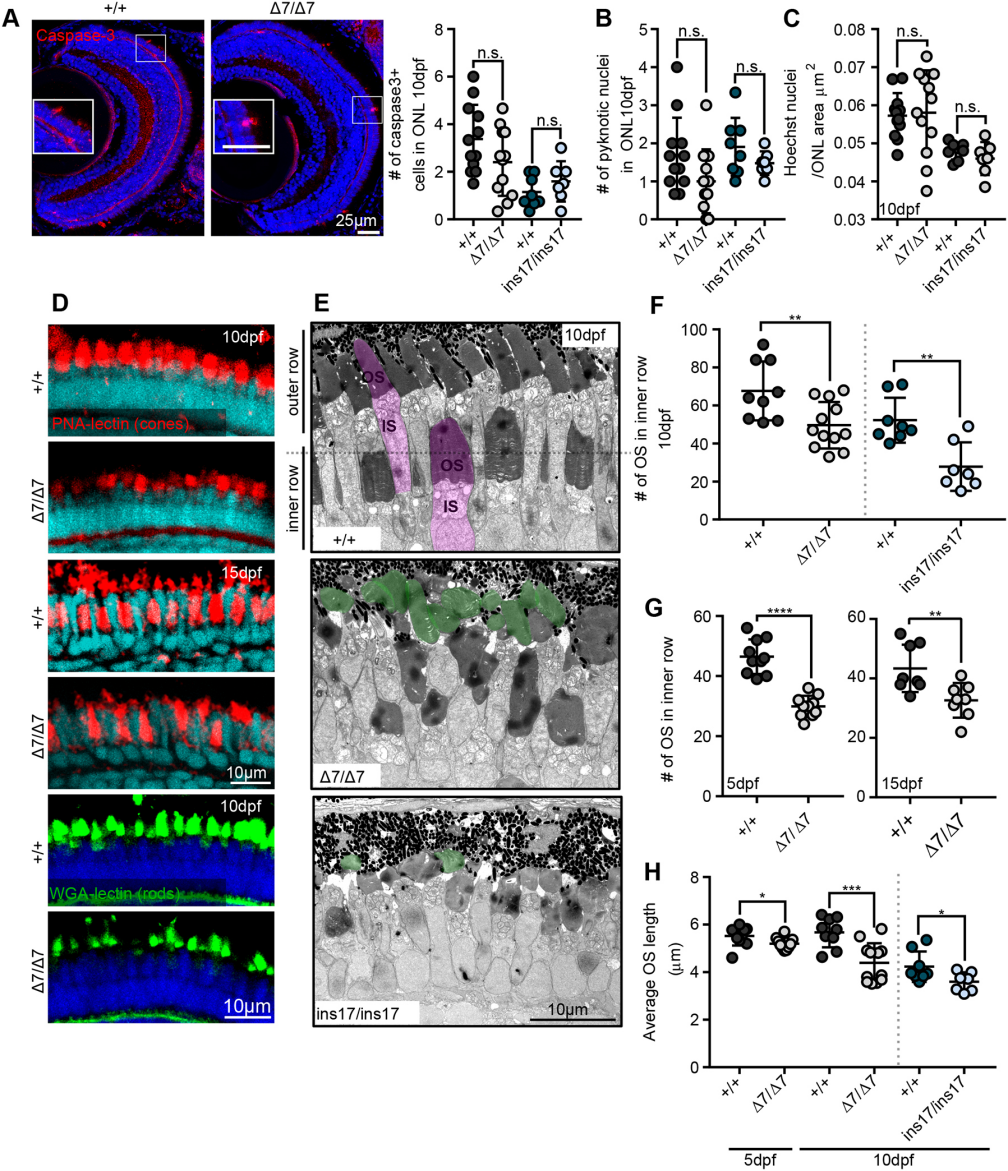
**Cone and rod photoreceptors show reductions in OS number and length that are not attributed to cell death or loss.** (A) Staining and quantification of the cell death marker, cleaved (activated) caspase-3, at 10 dpf in the ONL per section (+/+, 12 eyes) (Δ7, 12 eyes). (B) Quantification of the number of pyknotic nuclei in the ONL. (C) Photoreceptor nuclei (Hoechst) density/μm^2^ in the ONL at 10 dpf, measured from an average of 20 µm-thick sections. For B and C: +/+, 12 eyes; Δ7, 11 eyes; +/+, 8 eyes; ins17, 7 eyes. (D) Representative images of OS markers for cones (PNA lectin, red) and rods (WGA lectin, green) at 10 dpf and 15 dpf. (E) TEM images of the central retina of wild-type siblings (+/+) and Δ7 and ins17 mutants at 10 dpf. Magenta represents individual photoreceptor; green represents detached OS. (F,G) Quantification of the number of OSs in the inner row at 10 dpf (F), 5 dpf (G, left) and 15 dpf (G, right). (H) Quantification of the average length of all outer segments in the retina per section in wild-type siblings and Δ7 and ins17 mutants at 5 dpf and 10 dpf. Statistical analyses in A-C and F-H were performed using Student's *t*-tests (unpaired, two-tailed). n.s., not significant; *P≤0.05, **P≤0.01, ***P≤0.001, ****P≤0.0001. Number of eyes analysed for TEM data in E-G: 5 dpf: +/+, 10 eyes; Δ7/Δ7, 10 eyes; 10 dpf: +/+, 9 eyes; Δ7/Δ7, 12 eyes; +/+, 8 eyes; ins17/ins17, 7 eyes; 15 dpf: +/+, 8 eyes; Δ7/Δ7, 8 eyes. At least *n*=5 individuals were assessed for each immunostained marker. IS, inner segment; ONL, outer nuclear layer; OS, outer segment.

We next looked more closely at the photoreceptors. Owing to the association of USH1 clinical assessments with an RP ‘degenerative’ phenotype, we hypothesized that *pcdh15b* is not necessarily required for photoreceptor development but rather their maintenance over time and predicted there may be a loss of photoreceptors over time (El-Amraoui and Petit, 2014). However, using the red-green cone cell body marker, Zpr1, we were unable to observe abnormalities in our *pcdh15b* mutants (Fig. S3C). We examined cell death in the ONL by expression of cleaved (activated) caspase-3 and by the presence of pyknotic nuclei in 10 dpf retina and found no difference in the number of caspase3^+^ cells or pyknotic nuclei in the *pcdh15b* mutants compared to that of controls (Fig. 2A,B). In addition, the density of photoreceptor nuclei was not affected, suggesting that there was no ONL thinning (Fig. 2C). Therefore, these data suggest that death of photoreceptors was not significantly altered in mutants at the stages examined. In zebrafish, it is possible that enhanced regeneration of photoreceptors may partially underlie the apparent lack of ONL thinning in diseased or damaged states (Santhanam et al., 2020; Turkalj et al., 2021; Wan and Goldman, 2016), but, given that markers of cell death (apoptosis, pyknotic nuclei) are not observed in any time interval examined, we believe that this is unlikely.

Next, the OS phenotype previously observed in *Xenopus* knockdowns (Schietroma et al., 2017) led us to predict that although photoreceptors are still present, they may have abnormalities in their OSs. To examine this, we labelled for several different rod and cone OS markers. The cone OS marker, PNA lectin, showed a detectable decrease in size and number in *pcdh15b* mutants at 10 dpf onward (Fig. 2D). Additionally, the rod OS markers, Zpr3, RET-P1 and WGA-lectin, were all decreased and disorganized in Δ7 mutants (Fig. 2D; Fig. S3C). Whole-mount immunostaining in the 10 dpf retina additionally showed that Δ7 mutants predominantly lack proper rod OS staining, particularly in the peripheral retina, although cone OSs could still be broadly detected (Fig. S3E).

To quantify these differences more precisely, the histology of the photoreceptor layer was closely examined by transmission electron microscopy (TEM) and used to confirm the abnormalities in the OS. In the wild-type retina, the OSs in the photoreceptor layer are typically organized into two tiers with an ‘inner’ and ‘outer’ row (see Fig. 2E). The inner row is predominantly made of cone photoreceptor OSs and the outer row contains both rod and cone OSs (Branchek and Bremiller, 1984; Schmitt and Dowling, 1999). However, at 10 dpf, the zebrafish retina is mainly composed of cones, with fewer rods present (Branchek and Bremiller, 1984; Raymond et al., 1995). In *pcdh15b* Δ7 and ins17 mutant retinas at 10 dpf, the outer segments were disorganized and misshapen, and we observed a significant decrease (>40%) in the number of OSs in the inner row (cones) compared to that in wild-type siblings (Fig. 2E,F; Fig. S3G). In contrast, the number of OSs in the outer row was not significantly different (Fig. S3F). We observed that these specific inner row OSs decrease in the Δ7 mutants at both 5 dpf and 15 dpf, indicating that the OS numbers were affected early in photoreceptor development/maturation and were long lasting and not due to a developmental delay (Fig. 2G; Fig. S3G). Of the remaining OSs in the *pcdh15b* mutants, we also observed a decrease in average OS length compared to that of wild-type siblings, likely due to the apparent abnormal morphologies of the OS (Fig. 2H). Together, these results suggest that, in the early postembryonic period (up to 20 dpf), *pcdh15b* mutant photoreceptors exhibit prominent structural defects that precede any observable cell loss or thinning of the ONL. However, it is possible and likely that these structural OS defects eventually lead to photoreceptor loss classically seen in USH1 patients later in life.

### CPs are formed in *pcdh15b* mutant photoreceptors but have fewer contacts with the OS and are lost over time

**Fig. 3.**
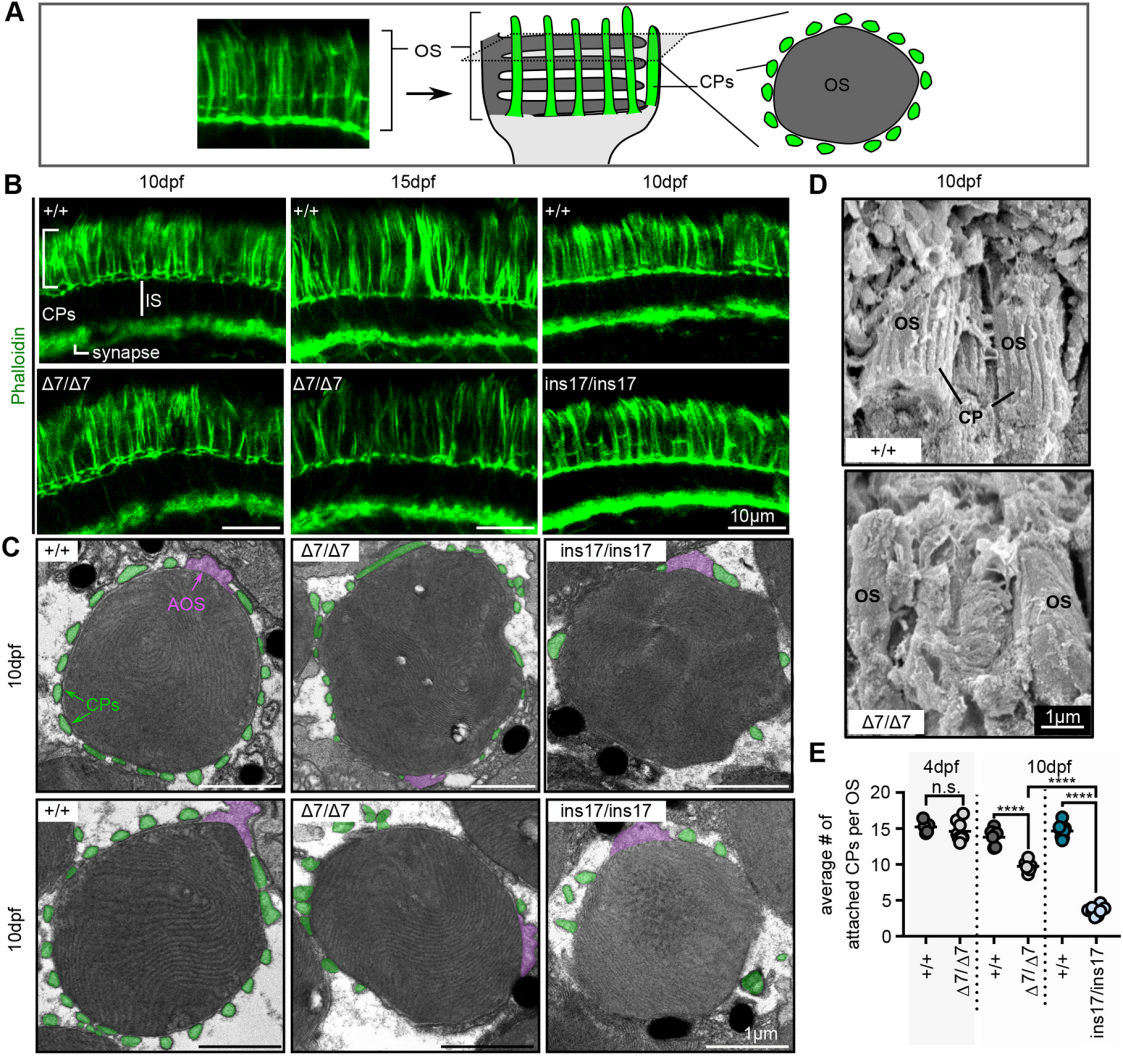
**CPs are formed in *pcdh15b* mutants but are weakly attached and reduced/lost over time.** (A) Schematic illustrating CPs surrounding the OS of photoreceptors. The F-actin stain, phalloidin, is used to label CPs. Transverse sections along the length of the photoreceptor show long CPs along their length, whereas horizontal cross-sections through the OS show OS surrounded by a ‘ring’ of CPs. In all images, CPs are coloured green. (B) Representative images of phalloidin staining in the photoreceptor layer in wild-type siblings (+/+) and Δ7 or ins17 mutants at 10 dpf and 15 dpf (*n*=8+ individuals for each genotype). (C) Representative TEM images of horizontal cross-sections through the OS in wild-type siblings and Δ7 and ins17 mutants. CPs (connected or disconnected to the OS) are coloured green; AOSs are coloured magenta. (D) Representative SEM images show CPs along the OS in wild-type siblings, which are absent/decreased in Δ7 mutants (5 eyes from *n*=5 individuals per genotype). (E) Quantification of the average number of connected CPs per OS in wild-type siblings and *pcdh15b* mutants at 4 dpf and 10 dpf. OSs quantified were sampled from the central retina in the ONL. Number of eyes analysed for CP counts: 4 dpf: +/+, 5 eyes; Δ7/Δ7, 8 eyes; 10 dpf: +/+, 6 eyes; Δ7/Δ7, 6 eyes; +/+, 8 eyes; ins17/ins17, 8 eyes. Statistical analyses in E were performed using Student's *t*-test (unpaired, two-tailed) for 4 dpf and one-way ANOVA for 10 dpf. n.s., not significant; ****P≤0.0001. AOS, accessory outer segment; CP, calyceal process; IS, inner segment; OS, outer segment.

Previous research, as well as our analysis, has shown that Pcdh15 is localized to the CPs in photoreceptors (Sahly et al., 2012). Furthermore, *pcdh15* knockdown in *Xenopus* results in reduction in the number of CPs (Schietroma et al., 2017). We utilized immunohistochemistry to label F-actin-rich CPs with phalloidin, as well as TEM and SEM to visualize CPs surrounding OSs (Fig. 3A) to determine how CPs were affected in our *pcdh15b* mutants. Surprisingly, we found that phalloidin-labelled CPs were still present in our Δ7 and ins17 mutants at 10 dpf, although we noticed a decrease in fluorescence intensity by 15 dpf in Δ7 mutants (Fig. 3B). This suggested that actin-filled CPs could still form in our *pcdh15b* mutants, although it remained unclear whether they formed adherent structures to the OS. To examine this further, we performed SEM on retinal tissue, and noticed a reduction in intact CPs surrounding the OS in our *pcdh15b* mutants compared to wild-type siblings (Fig. 3D). This suggests that CPs have fewer contacts with the OS and may be more prone to detachment. To test this, we visualized CPs by TEM in transverse and horizontal cross-sections. We observed that CPs in *pcdh15b* mutants were shorter in length and fewer in number compared to those in their wild-type siblings (Fig. S4A-C). We quantified the number of attached CPs per OS in horizontal sections from the central ONL and found that the number of attached CPs in mutants was comparable to that in wild type at 4 dpf, but that this number was significantly reduced by 10 dpf, particularly in the ins17 mutants (Fig. 3C,E). Interestingly, we also observed evidence of ‘detached’ CPs in the Δ7 and ins17 mutants (Fig. 3C; Fig. S4D). These data suggest that CPs in *pcdh15b* mutants are more prone to detachment and eventual loss but initially formed properly. CP loss occurs alongside the observed photoreceptor OS defects described earlier, but whether it contributes to the OS defects or is a consequence of them is difficult to distinguish.

### *pcdh15b* mutant retinas exhibit abnormal directional growth of outer segment discs

**Fig. 4.**
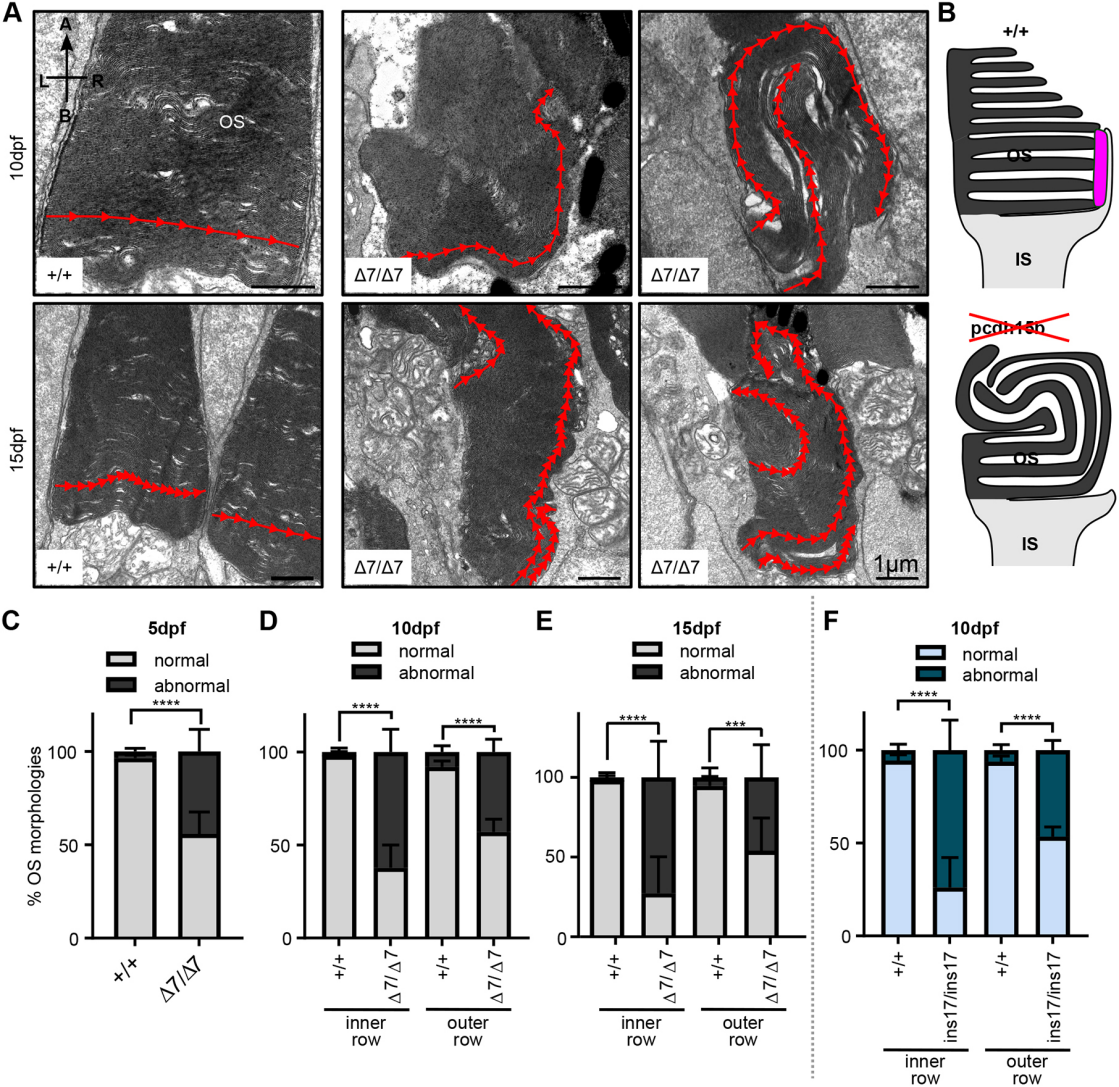
**Ultrastructure of OSs in *pcdh15b* mutant photoreceptors exhibit abnormal directional growth of discs.** (A) Representative TEM images of OSs of wild-type siblings and Δ7 mutants at 10 dpf and 15 dpf. Red arrow line shows disc direction. Directional arrows at the top left of the first image indicate positioning. (B) Schematic of the effects of *pcdh15b* mutation on the growth of the OS discs. Pcdh15 is shown in +/+ by the magenta-coloured shape. (C-F) Quantification of the percentage of ‘normal’ and abnormal OSs in wild-type siblings and *pcdh15b* mutants at different time points and mutant types (Δ7 and ins17). Statistical analyses in C-F were performed using Student's *t*-tests (unpaired, two-tailed). ***P≤0.001, ****P≤0.0001. OSs were analysed across the entire ONL for the 5 dpf and 10 dpf data, and the central retina for 15 dpf data. Number of eyes/OSs analysed: (B) 5 dpf: +/+, 10 eyes/2123 OSs; Δ7/Δ7, 10 eyes/1874 OSs; (C) 10 dpf: +/+, 9 eyes/1990 OSs; Δ7/Δ7, 12 eyes/2457 OSs; (D) 15 dpf: +/+, 6 eyes/320 OSs; Δ7/Δ7, 6 eyes/291 OSs; (E) 10 dpf: +/+, 8 eyes/1260 OSs; ins17/ins17, 7 eyes/956 OSs. A, apical; B, basal; IS, inner segment; L, left; ONL, outer nuclear layer; OS, outer segment; R, right.

To investigate the photoreceptor defects in more detail, we used TEM to analyse the ultrastructure of the OS discs. Previous *pcdh15* knockdown in *Xenopus* suggested two main OS phenotypes: (1) cone OS bending; and (2) basal overgrowth and bulging of rod OS (Schietroma et al., 2017). In contrast, we did not observe any obvious OS bending or enlarged bases of OSs (see Fig. 2D,E). However, there were clear defects in the canonical ‘cone’ and ‘rod’ OS shape (Fig. 2D,E). In wild-type sibling retinas, OS discs typically grow horizontally (left to right) and are bounded by the limits of CPs and adjacent photoreceptors. As OSs grow, newly formed discs grow at the base of the OS and result in linearly stacked discs making up the height of an OS (see Fig. 4A,B). In contrast, in *pcdh15b* mutants, OS discs grew in abnormal directions, leading to misshapen OSs, likely contributing to the observed decrease in OS length (Fig. 2H and Fig. 4A,B). Although growth tended to start perpendicularly in the mutants, continued growth was often seen apically and even basally orientated. It is important to note that abnormal directional growth occurred in OSs that still had contact with CPs, indicating that these growth defects can occur regardless of the presence of CPs. This abnormal growth and morphology occurred in a significantly high percentage of OSs in the retina (>50%) of both Δ7 and ins17 mutants and was observed as early as 5 dpf (Fig. 4C-F). The detection of this defect in the early retina suggests this is likely a developmental rather than degenerative defect. Interestingly, OSs in the inner row were more deformed (∼75%) than those in the outer row (50%), also suggesting that cones may be more prone to OS disc growth misregulation in these mutants (Fig. 4C-F).

### Outer segments detach and ‘float’ away from the photoreceptor cell body in *pcdh15b* mutants

**Fig. 5.**
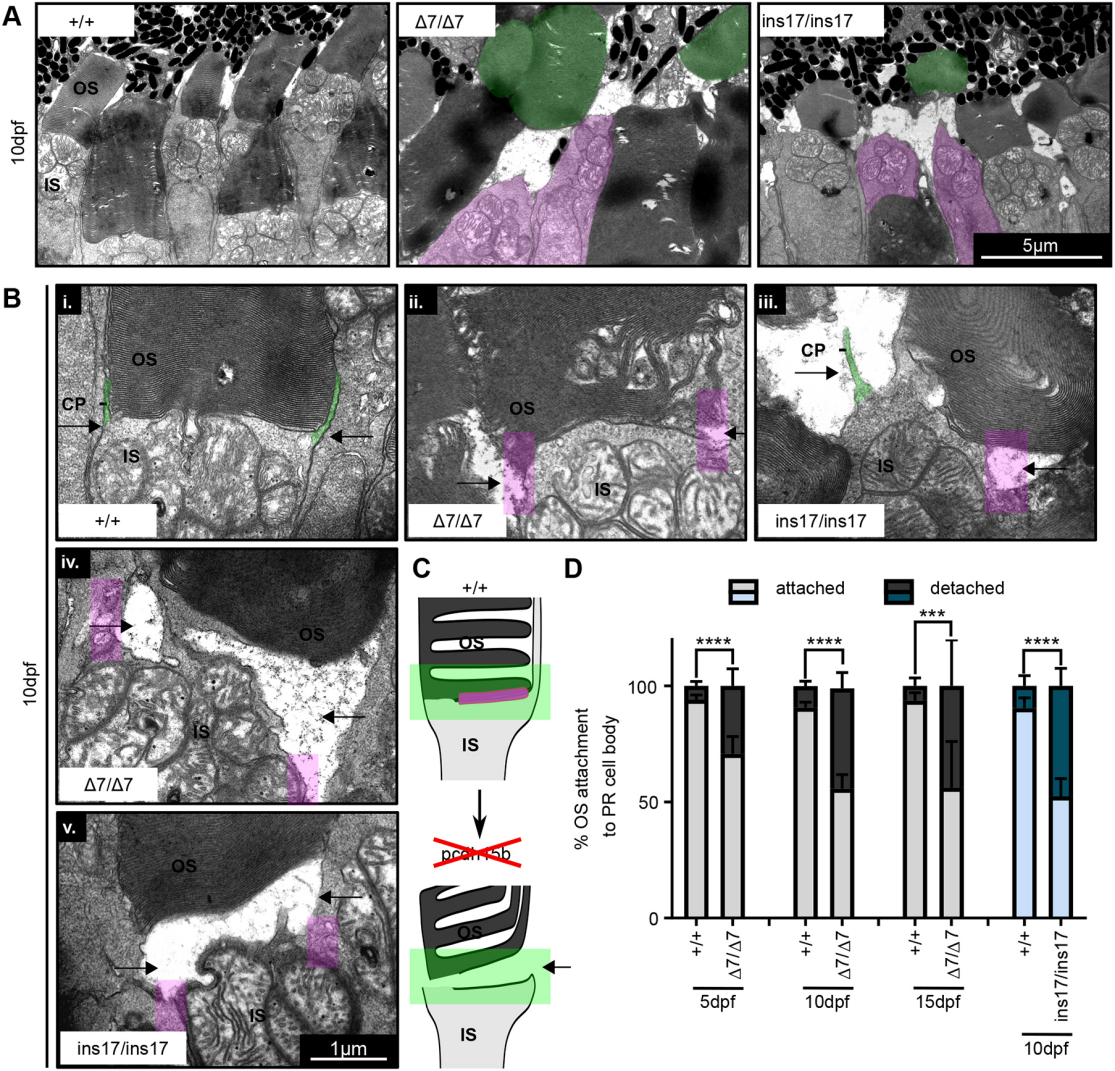
**OS detachment from the photoreceptor cell body is increased in *pcdh15b* mutants.** (A) Representative TEM images of OS detachment and floating OSs seen in *pcdh15b* mutants (Δ7 and ins17), compared to wild-type siblings. Magenta is used for IS with no OS; green is used for detached ‘floating’ OS. These phenomena are rarely observed in wild-type siblings. (B) Close-up TEM examples of IS-OS connection in photoreceptors of wild-type siblings and Δ7 and ins17 mutants, showing the process of detachment. CPs are visible as protrusions coming up from the IS (green) and attaching to the sides of the OS (arrows point to the sides). *pcdh15b* mutants (Δ7/Δ7 and ins17/ins17) show a range of affected connections (i-v), coincident with the loss of CP (magenta-coloured boxes) attachments on the sides (arrows). (C) Schematic of the OS detachment seen in *pcdh15b* mutants. The relevant Pcdh15 expression found at the OS base is highlighted in magenta. The green box highlights the IS-OS connection. (D) Quantification of the percentage of total identified photoreceptor IS/cell bodies with attached or completely detached/missing OSs across the entire ONL from 5 dpf to 15 dpf. Statistical analyses were performed using Student's *t*-test (unpaired, two-tailed). ***P≤0.001, ****P≤0.0001. Number of eyes/PRs analysed: 5 dpf: +/+, 10 eyes/1780 PRs; Δ7/Δ7, 10 eyes/1473 PRs; 10 dpf: +/+, 9 eyes/1844 PRs; Δ7/Δ7, 12 eyes/2290 PRs; 15 dpf: +/+, 6 eyes/334 PRs; Δ7/Δ7, 6 eyes/474 PRs; 10 dpf: +/+, 8 eyes/1253 PRs; ins17/ins17, 7 eyes/1087 PRs. CP, calyceal process; IS, inner segment; ONL, outer nuclear layer; OS, outer segment; PR, photoreceptor.

We were interested to know why there were decreased numbers of OSs, particularly in the inner row of the photoreceptor layer in *pcdh15b* mutants. Because our data suggested that there was no loss of photoreceptor cells, we reasoned instead that OSs were ‘missing’ from their photoreceptor cell bodies. Consistent with this interpretation, we commonly observed ‘floating’, detached OSs, especially in the outer row, significantly more frequently in mutant photoreceptors (Fig. 5A, green OS and magenta IS). We reasoned that OSs may be more prone to detachment from the photoreceptor cell body due to loss of Pcdh15b expression at the OS base. To observe this phenomenon, we analysed the IS-OS junction more closely by TEM. Compared to wild-type siblings, which have proper IS-OS attachments with visible CPs, Δ7 and ins17 mutant photoreceptors showed variable IS-OS connectivity. Some showed retracted or lost CPs, as well as loosening of the OS from the IS (Fig. 5Bi-iii) and even complete detachment (Fig. 5Biv,v,C). We quantified the percentage of photoreceptor cell bodies lacking any OS connection and found that Δ7 and ins17 mutants showed significantly elevated levels of detachment compared to wild-type siblings (close to 50%) at 10 dpf and onward (Fig. 5D). This detachment was seen as early as 5 dpf but to a lesser degree (25%), suggesting that phenotypic severity increases over time (Fig. 5D). Owing to the different observed stages of detachment, our data suggest that OSs start to loosen their connection to the IS, in conjunction with weakening contacts with CPs, followed by eventual detachment, resulting in ‘floating’ OSs commonly displaced into the outer row. The displacement into the outer row may be partly responsible for offsetting an observed decrease in OS number in this region, but likely explains the observed decrease in OSs present in the inner row. Detached OSs may be more prone to eventual loss as they are phagocytosed by retinal pigmented epithelium (RPE) (Strauss, 2005).

### Photoreceptor defects are developmentally acquired and progressively worsen over time in *pcdh15b* mutants

**Fig. 6.**
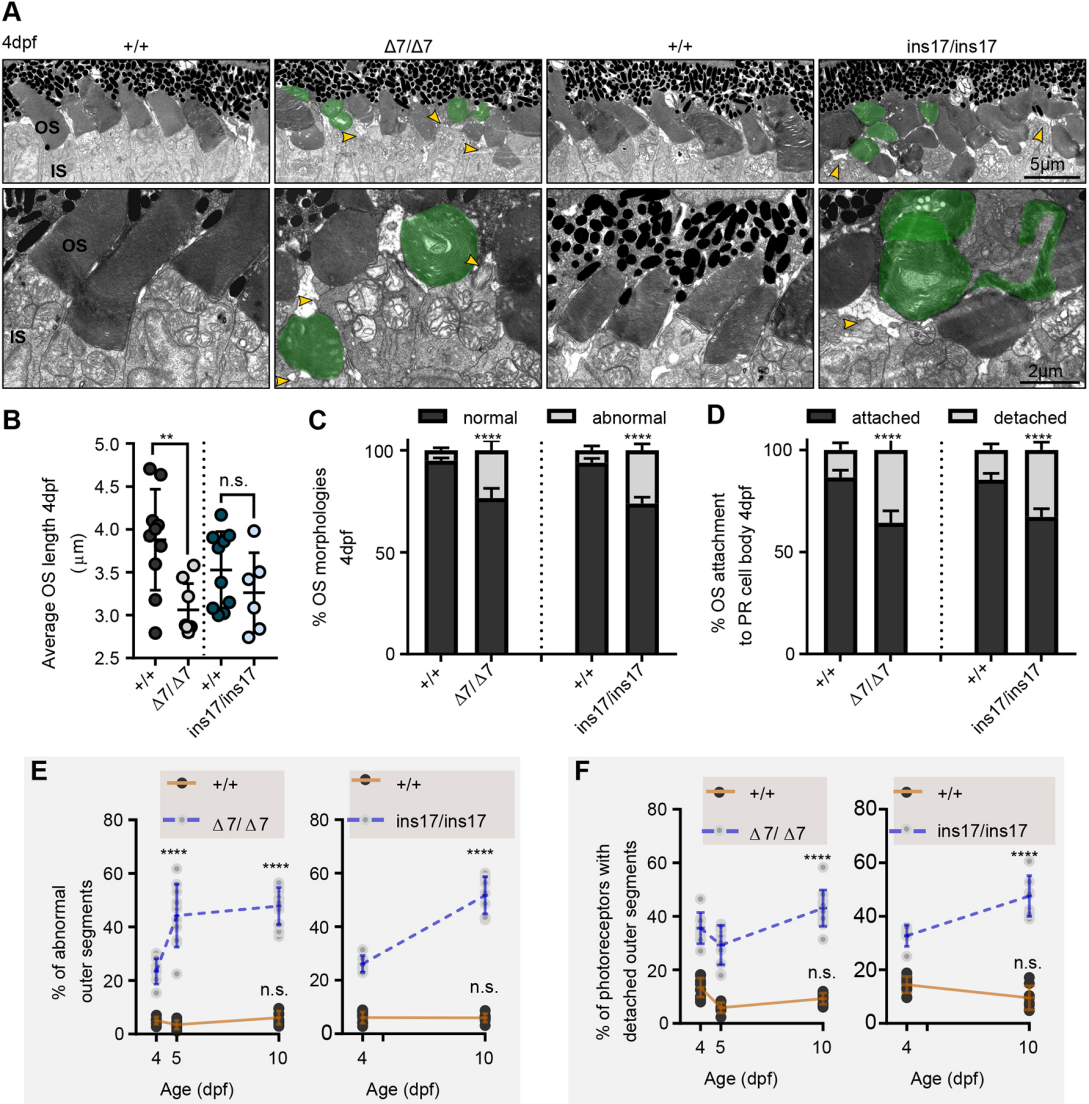
**Photoreceptors in *pcdh15b* mutants develop with defects that progressively worsen with age.** (A) Representative TEM images of the central ONL (top row) and OS (bottom row) in wild-type siblings, Δ7 and ins17 mutants at 4 dpf. Green represents deformed/abnormal OSs. Yellow arrowheads point to regions of disconnection between the IS and OS. (B-D) Quantification of the average length of all OSs in the retina per section (B), the proportion of OS morphologies (C) and the percentage of attached/detached OSs (D) in wild-type siblings, Δ7 and ins17 mutants at 4 dpf. The entire ONL was used for quantification. (E,F) Developmental trend of the percentage of abnormal OSs (E) and photoreceptors with detached OSs (F) in the retina from 4 dpf to 10 dpf in wild-type siblings, Δ7 or ins17 mutants. Statistical analyses were performed using Student's *t*-tests (unpaired, two-tailed) (B-D) and two-way ANOVA (E,F). n.s., not significant; **P≤0.01, ****P≤0.0001. Number of eyes/OSs analysed for 4 dpf data: +/+, 10 eyes/1528 OSs; Δ7/Δ7, 8 eyes/1412 OSs; +/+, 10 eyes/1829 OSs; ins17/ins17, 6 eyes/1328 OSs. IS, inner segment; ONL, outer nuclear layer; OS, outer segment.

Given that we observed photoreceptor defects as early as 5 dpf, we hypothesized that these defects were due to impaired development of photoreceptor structure rather than degeneration of initially properly formed photoreceptors. To test this hypothesis, we examined the ultrastructure of 4 dpf photoreceptors, representing a time point immediately after their differentiation and into their maturation phase of development. In zebrafish, differentiation of photoreceptors begins at ∼2.3 dpf, with the first OS appearing at ∼2.5 dpf and experiencing the greatest growth until 5 dpf (Branchek and Bremiller, 1984; Crespo and Knust, 2018; Sukumaran and Perkins, 2009). Therefore, we chose to examine a time point between the initial OS maturation and growth (i.e. 4 dpf) and found similar defects in the morphology and organization of photoreceptors to that observed in older retinas (Fig. 6A). We also noticed a slight decrease in the length of OSs of Δ7 and ins17 mutants compared to that of wild-type siblings (which is even more apparent at later stages previously described), indicating developmental OS growth defects (Fig. 6B). Importantly, we still observed a large percentage of abnormal OS discs (∼20-25%) (Fig. 6C) and photoreceptor cell bodies with missing or detached OSs (∼35-40%) at 4 dpf in our Δ7 and ins17 *pcdh15b* mutants (Fig. 6D). Combining our data from all the different time points analysed, we noticed an intriguing developmental trend: the mutants displayed significant OS abnormalities and detachment immediately following differentiation that worsened with time (Fig. 6E,F). These findings alter our current view that USH1 is primarily a degenerative condition by suggesting that photoreceptors may instead develop abnormally with worsening morphological consequences over time as the eye continues to mature.

### Exposure to different light intensity can attenuate or exacerbate photoreceptor defects in *pcdh15b* mutants

The defects we observed indicate that the photoreceptor OS is severely affected in *pcdh15b* mutant retinas. However, we wondered whether different environmental light conditions could attenuate or exacerbate these defects. This is because although USH mouse and zebrafish models show mild to no retinal phenotype, increased light exposure has been shown to result in heightened damage in photoreceptors of mutants (Dona et al., 2018; Peng et al., 2011; Trouillet et al., 2018; Wasfy et al., 2014). In addition, light-dark cycles are necessary for OS turnover (Besharse et al., 1977a,b), and, in the zebrafish retina, changes in this cycle have been seen to affect the growth of OSs (Crespo and Knust, 2018). We hypothesized that because OS disc growth and basal OS connection to the IS are defective in the mutants, the severity of these phenotypes is related to continuous OS turnover and therefore could be modulated by light exposure. To answer this question, we exposed our developing zebrafish larvae to either complete darkness (0 lux) or a normal 14 h-10 h light-dark cycle with 5 h periods of heightened bright light (3000/6000 lux) daily from 6 dpf to 10 dpf (Fig. S5A,B). Interestingly, darkness attenuated the severity of the photoreceptor defects, whereas bright-light conditions exacerbated the defects (Fig. S5A,B). In particular, the number of OSs detected in the inner row was reduced further in bright-light conditions compared to dark conditions in Δ7 mutants (Fig. S5C). However, this was not a consequence of elevated cell death/ONL thinning in the photoreceptor layer in mutants under bright light (Fig. S5D,E).

**Fig. 7.**
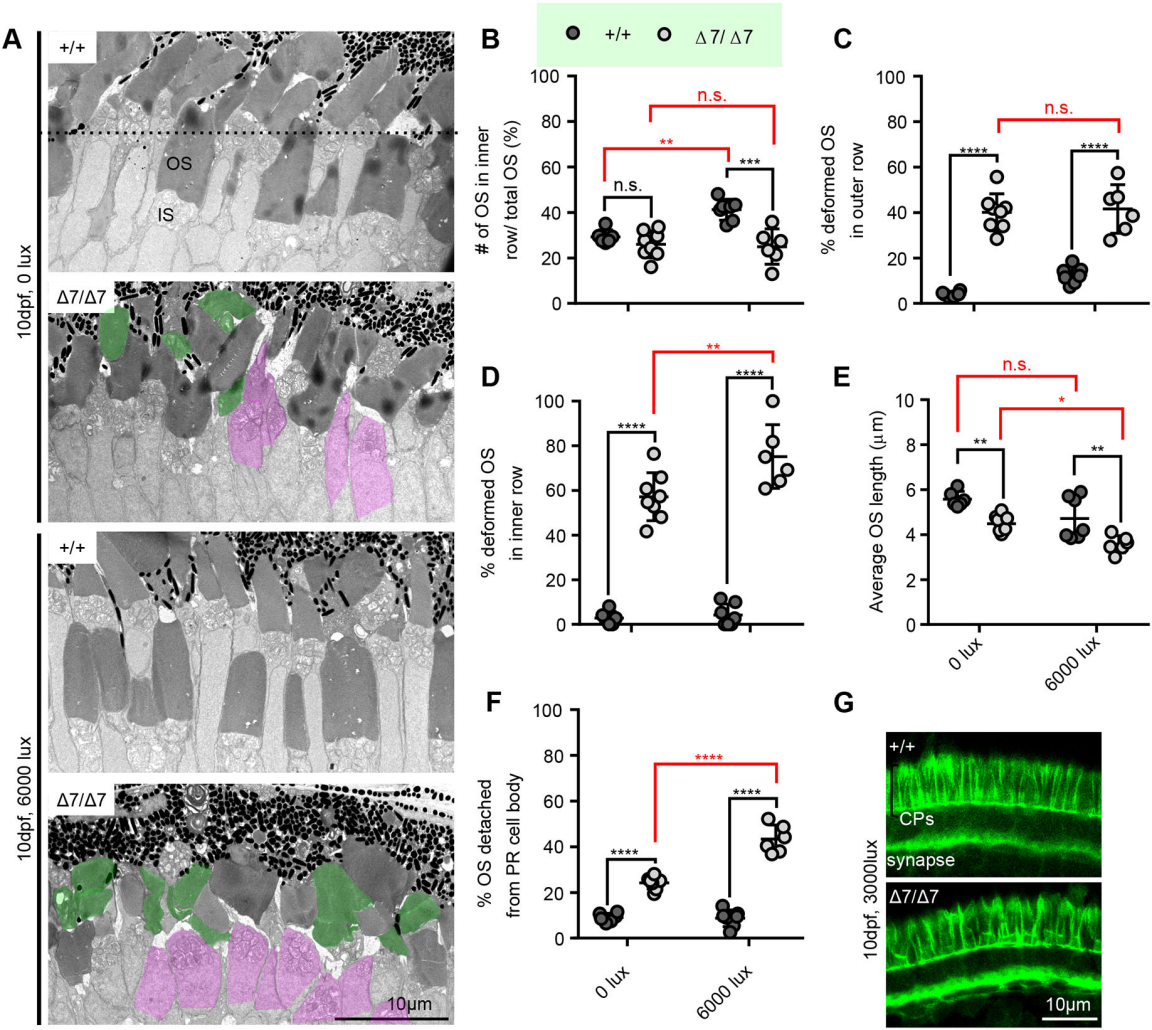
**Differential light exposure in *pcdh15b* mutants attenuates or exacerbates photoreceptor defects.** (A) Representative TEM images of wild-type sibling and *pcdh15b* mutant ONL at 10 dpf grown under 0 lux (dark) and 6000 lux (bright) light conditions. The dotted line in the top image separates the inner and outer row. ISs with detached OSs are coloured magenta. Deformed OSs are coloured green. (B-F) Quantification of the percentage of total OSs found in the inner row (B), the percentage of deformed OSs in the outer row (C) and the inner row (D), the average OS length (E), and the percentage of total identified photoreceptor IS/cell bodies with completely detached/missing OSs (F) across the retina photoreceptor layer under different light conditions in wild-type siblings and *pcdh15b* Δ7 mutants. All graphs in B-F use the same labelling as in B to distinguish between +/+ and Δ7/Δ7. *x*-axis labels are the same and seen at the bottom in E and F. (G) Representative phalloidin staining in the ONL, labelling CPs, at 10 dpf when exposed to 3000 lux bright light (+/+, 7 eyes; Δ7/Δ7, 7 eyes). 0 lux measurements were taken across the entire ONL, and 6000 lux measurements were taken only in the central ONL. Statistical analyses in B-D and F were performed using two-way ANOVA. Statistical tests in E were performed using one-way ANOVA. n.s., not significant; *P≤0.05, **P≤0.01, ***P≤0.001, ****P≤0.0001. Numbers of eyes/OSs analysed: 0 lux: +/+, 6 eyes/1239 OSs; Δ7/Δ7, 8 eyes/1623 OSs; 6000 lux: +/+, 7 eyes/541 OSs; Δ7/Δ7, 6 eyes/409 OSs. IS, inner segment; OS, outer segment.

We examined the differential light-treated photoreceptors in more detail by TEM and made several interesting observations (Fig. 7A). First, although we found that the number of inner row OSs was reduced in each condition, when normalized to the amount of total OSs in both rows, darkness eliminated this decrease, whereas this effect was enhanced in bright-light conditions, indicating that bright light exacerbates inner OS loss in mutants (Fig. 7B). We additionally found that bright light enhanced the percentage of deformed OSs in Δ7 mutants, specifically in the inner row, suggesting a strong effect on cone photoreceptors (Fig. 7C,D). This enhanced deformity likely contributes to the exacerbated decrease in average OS length we observed under bright-light conditions only in the mutants (Fig. 7E). The strongest effect we observed was an almost doubling of OS detachment from the photoreceptor cell body/IS in Δ7 mutants under bright-light conditions compared to dark conditions (Fig. 7F). Interestingly, phalloidin-labelled CPs were largely still present in mutants under bright-light conditions, although they looked more disorganized (Fig. 7G). However, by TEM, we did notice a decrease in intact CPs (Fig. S5F). This suggests that differential light exposure can reduce or enhance *pcdh15b*-associated retinopathic phenotypes.

### Increased disorganization of photoreceptor ribbon synapses in *pcdh15b* mutants

**Fig. 8.**
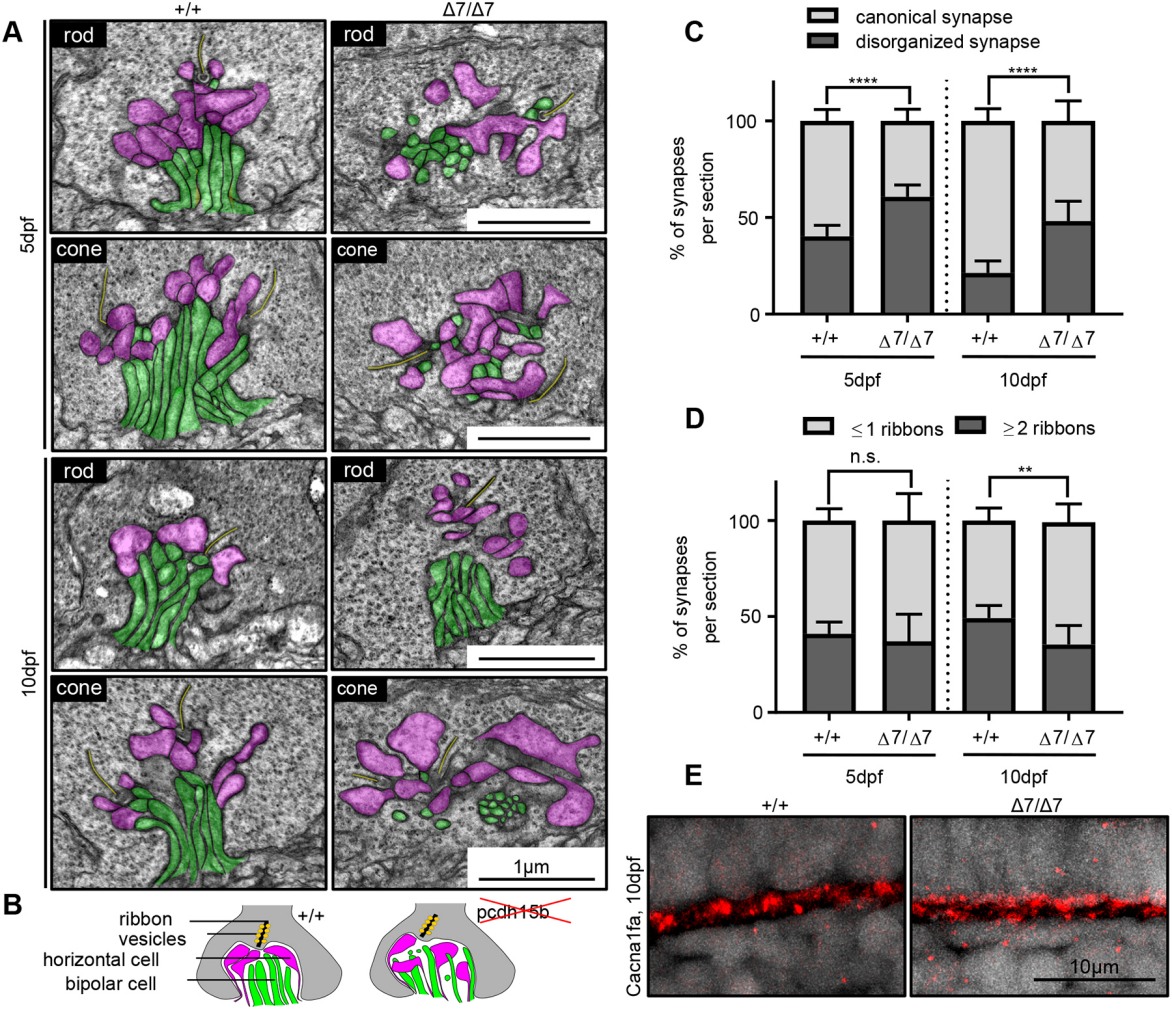
**Photoreceptor ribbon synapses show increased disorganization in *pcdh15b* mutants.** (A) Representative TEM images of photoreceptor ribbon synapses from both rod and cone terminals in wild-type siblings and *pcdh15b* Δ7 mutants at 5 dpf and 10 dpf. Bipolar cell innervations (green) and horizontal cell innervations (pink) into photoreceptor termini and ribbon synapses (yellow) are highlighted. (B) Schematic of synaptic structure in wild-type and *pcdh15b* mutants. Magenta, horizontal cell processes; green, bipolar cell processes. Ribbons are shown as a black line, surrounded by yellow-coloured vesicles. (C) Quantification of the percentage of synapses that display a canonical or disorganized synapse organization in wild-type siblings and Δ7 mutants at 5 dpf and 10 dpf. (D) Quantification of the percentage of synapses that contain one or less ribbons (usually associated with rod termini), or two or more ribbons (usually associated with cone termini), in wild-type siblings and Δ7 mutants at 5 dpf and 10 dpf. (E) Representative images of calcium channel 1.4_v_ staining (Cacna1fa) in the outer plexiform layer, at photoreceptor termini (*n*=5+ individuals for each genotype). Statistical analyses in C and D were performed using Student’s *t*-tests (unpaired, two-tailed). n.s., not significant; **P≤0.01, ****P≤0.0001. Number of eyes/synapses analysed: 5 dpf: +/+, 10 eyes/620 synapses; Δ7/Δ7, 10 eyes/593 synapses; 10 dpf: +/+, 8 eyes/201 synapses; Δ7/Δ7, 8 eyes/192 synapses. Synapses used in quantification were sampled from the central ONL.

Aside from the OS, localization of Pcdh15 and other USH-related proteins has been observed in the synapse of inner hair cells and photoreceptors (Reiners et al., 2005; Zallocchi et al., 2012). However, few studies have investigated the role of USH proteins in these specialized ribbon synapses. Of the USH-related proteins, type 3 protein, Clarin1, has been most strongly linked to the synapse and to proper synaptic structure in auditory hair cells (Dulon et al., 2018). Given that *Pcdh15* and *Cdh23* have been linked to the actin cytoskeleton, we hypothesized that *pcdh15b* may play a role in establishing synaptic organization, although it has never been investigated (Cosgrove and Zallocchi, 2014; Dulon et al., 2018). To analyse this, we used TEM to observe synaptic structure in the central retina at 5 dpf and 10 dpf (Allwardt et al., 2001; Schmitt and Dowling, 1999). We found that synapses contained all necessary synaptic ribbon components, including anchored ribbons, tethered vesicles, bipolar and horizontal cell innervation, but that the synapses were increasingly disorganized in Δ7 mutants at 5 dpf and 10 dpf (Fig. 8A-C). Unlike canonical synapses of rods and cones, characterized by medial bipolar cell innervations surrounded by larger horizontal cell innervations, disorganized synapses commonly had displaced and dispersed innervations (Fig. 8A,B). Additionally, we noticed there was a significant decrease in the number of synapses with two or more ribbons at 10 dpf in Δ7 mutants (Fig. 8D). Rod synapses are generally characterized by having one ribbon, whereas cones contain multiple ribbons. Therefore, in addition to evidence of disorganized synapses, there appears to be an overall decrease in the percentage of synapses with cone-like features. We wondered whether the disorganization would be reflected in the presence of synaptic proteins and therefore assayed the localization of the photoreceptor synapse calcium channel, Ca_v_1.4 (Cacna1fa) (Jia et al., 2014). At 10 dpf, we observed diffuse, smaller puncta staining in Δ7 mutants compared to wild-type siblings (Fig. 8E). Given the localization of Pcdh15 expression in the photoreceptor synapse of zebrafish, Pcdh15b may interact with synaptic components that connect to F-actin filaments, but the exact mechanism is unclear. Overall, our data suggest that *pcdh15b* is necessary to maintain photoreceptor synaptic organization.

## DISCUSSION

We present one of the first genetic animal mutants for *PCDH15* that displays a severe, early retinopathy and suggests that zebrafish could be a useful model for USH1F-associated retinal phenotypes. Our findings demonstrate that *pcdh15b* function is required in three main subcellular compartments in photoreceptors: CPs, the IS-OS junction and the synapse (Fig. S6A-D). Specifically, we found that, in our mutants, CPs maintain fewer contacts with OS, resulting in OS disc growth misdirection, especially in cone photoreceptors. In agreement with Schietroma et al. (2017), our data suggest that *pcdh15b* attaches CPs to OSs, but we note that *pcdh15b* does not appear to be required for initial CP formation, but rather to stabilize the connection of CPs to OSs over time (Fig. S6B). Second, we found that photoreceptor structural integrity was compromised, resulting in severe detachment of OSs from the IS. This suggests that *pcdh15b* expression at the IS-OS junction functions to anchor the basal discs to the plasma membrane of the IS (Fig. S6C). Lastly, we found disorganization of synaptic components, suggesting that *pcdh15b* is required for proper synaptic architecture (Fig. S6D), possibly by linking synaptic components to the F-actin network. Interestingly, all these effects were found as early as 4 dpf, suggesting that these defects are developmentally acquired. We also show that *pcdh15b* zebrafish mutants have selective defects in photoreceptors of the retina but not cells of the ear, providing the unique opportunity to study retinal specific defects of USH1F. Nonetheless, one of the limitations of this mutant is that they are larval lethal, which inevitably limits longer-term studies. Understanding what additional systemic defects occur that lead to death or the creation of conditional retinal knockouts could be useful if longer-term studies are desired.

### *pcdh15b* function in CPs

Our current model refines the previous model proposed for Pcdh15 and other associated USH1 proteins at CPs (Sahly et al., 2012; Schietroma et al., 2017). The previous model supported by *Xenopus* studies suggested that USH1 proteins function in CPs to stabilize and prevent bending of cone OSs and limit basal OS disc growth in rods (Schietroma et al., 2017). We do not observe cone OS bending, but instead notice extensive abnormal directional growth of OS discs in rods, and especially in cones, given the highly cone-dominant zebrafish retina. Thus, we posit that the main function of USH1-associated proteins in CPs is to link to the ends of OS discs to control/limit their growth. It is possible that the differences seen between *Xenopus* and zebrafish may, in part, be due to differences in methods (knockdown versus mutant analysis, or timing of analysis), inherent differences in photoreceptor density and composition between *Xenopus* (rod dominant, similar to mice) and zebrafish retinas (cone dominant) (Gábriel and Wilhelm, 2001; Noel et al., 2021; Röhlich and Szél, 2000), and the partial functional compensation of *pcdh15a* in zebrafish retinas. However, evidence for a role for *pcdh15a* in the retina is limited (Seiler et al., 2005), and more research is required.

Interestingly, studies of a mutation in a retinal-specific gene, protocadherin21 (*Pcdh21*), which belongs to a subset of cadherins structurally similar to *Cdh23* and *Pcdh15* (Elledge et al., 2010), report morphological defects that are similar to those reported here. PCDH21 is expressed at the base of photoreceptors, where it links open lamellar evaginations of discs in rods and cones to the opposing IS membrane (Burgoyne et al., 2015). Knockout of *Pcdh21* in mice results in abnormal disc organization and morphogenesis, similar to our mutants, and leads to eventual photoreceptor degeneration (Rattner et al., 2001). Additionally, loss of prominin-1, a known PCDH21-interacting protein expressed at the base of OSs, also results in OS discs displaying abnormal directional growth, similar to our mutants (Lu et al., 2019; Yang et al., 2008). With this model, the differences between rod and cone structure can account for the increased severity we notice in cones. Rods are encapsulated by an outer plasma membrane that only leaves basally located newly forming lamella exposed. Conversely, cone OS discs are made entirely of open lamella evaginations and only have CPs to tether onto for structural stability (Bujakowska et al., 2017; Goldberg et al., 2016; Steinberg et al., 1980). It is notable that mutations in human *PCDH21* are associated with cone-rod dystrophy and retinal degeneration (Henderson et al., 2010; Ostergaard et al., 2010), but an association of PCDH15 or other USH1 proteins with cone-rod dystrophy is, however, unclear. Pcdh15-attached CPs must be especially important for cone OS disc growth, explaining why cones are more prone to deformation in our *pcdh15b* mutants. It is not clear why only a subset of OSs is affected in our mutants (i.e. 50-75%), but it seems plausible that, in addition to potential rod and cone differences, the phenotype is age dependent as we see defects worsen over time. It is also possible that there is some, perhaps variable, compensation by *pcdh15a*.

According to this model, *pcdh15b* is functioning similarly in the retina, as *pcdh15a/Pcdh15* in the inner ear, to ‘adhere’ structures together. However, in the ear, the physical length and elasticity/tension created by PCDH15-CDH23 links in stereocilia and in response to deflection has a clear functional role in mechanotransduction (Dionne et al., 2018; Jaiganesh et al., 2018). Whether photoreceptors require molecules with these physical properties is unclear. Furthermore, it is not known whether the Pcdh15-Cdh23 interaction is conserved in the zebrafish retina, owing to the lack of *cdh23* expression observed in photoreceptors (Glover et al., 2012). If not, is it possible that Pcdh15 forms a homophilic interaction to mediate adhesive connections in the structures of the photoreceptor? This is unknown and needs to be further tested biochemically, but combinations of alternatively spliced Pcdh15 molecules that form a pseudo-heterophilic tip link may be possible (Narui and Sotomayor, 2018). Another question raised is why elasticity and tension created by these molecules would be required in photoreceptors. It is possible that the dynamic nature of apical shedding and basal growth of newly formed discs in photoreceptors (Goldberg et al., 2016; Willoughby and Jensen, 2012) require elastic forces to maintain structural integrity. This may explain why different light exposures, which may affect the dynamic growth of OSs, change the severity of the phenotype. Studies mimicking known mutations that reduce the elasticity of the Pcdh15 while maintaining other Pcdh15 structural properties (Bartsch et al., 2019) in the retina can be examined. Testing whether similar defects to our mutants are observed or not with these specific mutations can determine whether these physical properties are important for vision. Moreover, our western blot analysis, along with other studies (Zallocchi et al., 2012), indicates that multiple isoforms of different sizes are expressed in the retina, suggesting the possibility that the retina requires some Pcdh15 molecules with different properties.

### *pcdh15b* depletion causes loss of OS prior to overt photoreceptor cell death

Our results suggest that *pcdh15b* has additional functions in the photoreceptor. First, *pcdh15b* appears to be necessary to connect the base of the OS to the IS. Loss of this connection may put additional strain on the connecting cilium and axoneme, resulting in eventual breakage and complete detachment. Previous USH1 studies exclusively looked for retinal thinning or markers of cell death to establish a retinal phenotype in animal models (Glover et al., 2012; Libby et al., 2003; Peng et al., 2011; Wasfy et al., 2014; Williams et al., 2009). Although it is possible, and even likely, that OS loss leads to cell death, our results suggest that OS loss precedes any observable loss in photoreceptors. Intriguingly, this may suggest that early treatment focused on stabilizing OS biogenesis, or initiating it *de novo*, rather than replacement of lost photoreceptors, may be a more beneficial approach for USH1 therapy before widespread death (Horton et al., 2015).

How does *pcdh15b* loss of function lead to OS defects? Our data suggest that, unlike what was previously proposed, CP loss is not the primary defect. This is because CPs appear to form and are only observed to decrease by 10 dpf, a time point at which other defects are already evident. Therefore, we propose a model in our mutants in which CPs are formed normally, but they are improperly connected (or lack connection) to OS discs. This allows the OS discs to grow beyond or away from CP boundaries as they fail to form a proper disc-CP attachment over time (Fig. S6E). Eventually the OS discs also lose attachment to the IS, as OSs turn over and put additional strain on the connecting cilium/axoneme to break off entirely, leaving CPs to eventually retract (Fig. S6E).

### *pcdh15b* and synapse organization

Our results also suggest that *pcdh15b* function is required at the photoreceptor synapse. Expression data of USH1 proteins has shown that all proteins, albeit possibly as different and even shorter isoforms, are present at photoreceptor synapses, suggesting that it is a main site of USH1 function (Reiners et al., 2006). Both the long full-length and shortened isoforms (likely missing the extracellular domain) of Pcdh15 have been described in the synapse (Alagramam et al., 2011), and our data suggest that the shorter isoforms are likely still expressed in our mutant. Therefore, although we do see clear organizational defects, complete disruption of *pcdh15b* function in the synapse may not be fully reflected from our mutant.

Clarin-1, a type 3 USH protein, has the strongest functional link to synapses in hair cells so far. Loss of clarin-1 results in delays in synaptic maturation (Ogun and Zallocchi, 2014; Zallocchi et al., 2012) and in abnormal clustering of calcium channels, Ca_v_1.3, at the active zones of ribbons of inner hair cells, leading to lower calcium efficiency of exocytosis (Dulon et al., 2018). Disruption and loss of organized synaptic actin networks due to clarin-1 loss are also observed (Dulon et al., 2018), suggesting that actin maintains tight spatial organization of Ca_v_1.3 necessary for excitatory transmission (Vincent et al., 2015). Similar connection to synaptic activity and synaptic F-actin has been observed for the USH2 gene, whirlin (Jepson et al., 2014; Kersten et al., 2010). In photoreceptors, tight spatial control of Ca_v_1.4 beneath the synaptic ribbon is regulated by actin (Mercer et al., 2011). It has also been previously observed that Clarin-1 physically interacts with Pcdh15a in zebrafish hair cells (Ogun and Zallocchi, 2014). Therefore, in agreement with previous data, our results link *pcdh15b* in zebrafish photoreceptors to synaptic organization and Ca_v_1.4 localization, which may impact synaptic function. This disorganization in the mutant occurs early and occurs as the synapse is developing (Fig. S6E). However, the exact protein interactions of Pcdh15b with other USH1 or other synaptic components responsible remain poorly understood.

### Clinical features of RP associated with *PCDH15* mutation

Although our mutants display a severe retinopathy, the heightened severity in cones is atypical for RP, the primary retinal prognosis in USH patients. In addition, we show that the defects can be exacerbated in cones, but not rods, under bright light. However, rods clearly show defects in our mutants as well. To date, no cases associated with *PCDH15* mutations have been described as a cone-rod dystrophy. Clinical data among USH1 cases though are variable in their progression, and some cases have been associated with colour blindness-related defects, including tritan defect and severe dyschromatopsia (Khateb et al., 2019; Mrugacz et al., 2010). In addition, studies of albino harmonin and sans knockout mice show primary degeneration of cones (Trouillet et al., 2018). A recently identified USH-like causative gene, *CEP290*, is also associated with cone-rod dystrophy (Fuster-García et al., 2018).

Our data suggest that *PCDH15* and other USH1 gene mutations may present progression of features atypical of RP. The early onset of photoreceptor defects in our mutants also highlight that causes of RP for USH1 may be due to improper development of photoreceptor OS and synapses rather than later-onset degeneration in young children/adolescents typically seen for USH1-related RP. These phenotypes may prove useful in ‘early’ diagnosis of USH1-associated retinal defects before the onset of blindness. Furthermore, it might be possible to utilize our *pcdh15b* mutants in chemical screens to identify known or novel compounds that ameliorate the photoreceptor defects. Lastly, the retinal-specific consequences found in our *pcdh15b* mutants, and not in *pcdh15a* mutants (Seiler et al., 2005), may provide insight into why particular mutations in *PCDH15* are only associated with non-syndromic deafness, whereas others lead to USH1 (Ahmed et al., 2008).

## MATERIALS AND METHODS

### Zebrafish husbandry

All strains, including generated mutants, used in this study were derived from the wild-type-AB (WT-AB) zebrafish strain background obtained from the Zebrafish International Resource Center (ZIRC) and maintained at our facility at the University of Toronto. *pcdh15b* mutants generated in-house (see ‘CRISPR targeting of *pcdh15b* and genotyping’ section) were kept as heterozygous adults of both male and female sex for breeding, and mutant lines were outcrossed for at least two generations before being used for experiments. Unless indicated, all larvae used in the study were the result of in-crossing verified heterozygous male and female adults. All embryos/larvae used for further analysis were genotyped to separate wild-type, heterozygous and homozygous mutant genotypes from each other. Only mutants verified by genotyping were used for further analysis and siblings genotyped as wild type (+/+) were used as matched controls. In some cases, WT-ABs obtained from crossings of wild-type adults were also used as additional controls.

In all cases, embryos were collected and grown in a 28°C incubator, in a 100 mm-wide Petri dish containing 40-100 embryos, in the dark until 6 dpf. In most cases, after 6 dpf, the larvae were transferred to a 28°C-maintained animal facility to receive a 14 h-10 h light-dark cycle (unless in different light treatment groups) and fed 0 size food (Gemma Micro 75 zebrafish, Skretting) twice daily (morning and evening), with water changes twice a day. They were then grown to their desired age. If grown past 10 dpf, they were additionally fed brine shrimp in the afternoon. The sex of larvae used in the study is unknown because it is not possible to identify sex of zebrafish at this stage. Animals were treated in accordance with the regulations on animal experimentation established by the Canadian Council on Animal Care. The experimental procedures were approved by the University of Toronto Animal Care Committee.

### CRISPR targeting of *pcdh15b* and genotyping

CRISPR-Cas9 genome-editing technology was used to generate mutants for the *pcdh15b* gene in the WT-AB zebrafish background following a protocol previously described (Olsen et al., 2016). A gRNA targeting the 7th exon of the *pcdh15b* gene with the following sequence, GGGGCTGTTGACATCGATGA, was used to generate mutant founders (Varshney et al., 2013). Briefly, 200 pg gRNA (transcribed directly off a long oligonucleotide containing a T7 promoter) and 300 pg Cas9 mRNA (transcribed from pT3TS-nCas9n plasmid (Addgene #46757, RRID: Addgene_46757) were co-injected into the cell of a one-cell-staged embryo and grown to adulthood. The adult founders (F0) were outcrossed and assessed for germline transmission. Those with progeny containing mutations were grown to adulthood (F1) and genotyped by Sanger sequencing for mutations. Two were kept for further analysis, including a 7 bp deletion strain (known as Δ7; ZFIN: uot14) and a 17 bp insertion strain (known as ins17; ZFIN: uot15). These fish were further outcrossed (F2) and then in-crossed (F3) to obtain homozygous mutants; however, these were not viable as adults. Therefore, heterozygous adults were used for further breeding (see Results). Embryos/larvae used in this study are therefore F4 or older.

All embryos/larvae used in this study were subjected to genotyping. The Δ7 mutants were genotyped by PCR followed by restriction enzyme digestion by ClaI due to the mutation resulting in loss of a ClaI digestion site (which was confirmed by Sanger sequencing). The ins17 mutants were genotyped by Sanger sequencing because the sequence retains the ClaI digestion site. At least 30 or more larvae were genotyped per clutch from multiple clutches for the different experiments. The following primers were used for PCR: F, 5′-AAGAAAGAAAGAACGAAAGAAAGAAA-3′ and R, 5′-GATGGATGGCCATTTGAGAG-3′.

### RT-qPCR

Heterozygous fish were crossed, and embryos were collected. At 10 dpf, dissected eyes from individuals (from Δ7 heterozygous crossings) were homogenized into Trizol (Invitrogen, 15596018), while the body was used for genotyping. The Trizol solution from individuals were placed at −80°C until genotyping was completed. This was done for greater than 50 individuals from three different clutches (total 150+ embryos). Genotyped embryos were grouped into wild-type siblings, heterozygous and homozygous mutants, yielding three groups from three different clutches (three biological replicates). The RNA was extracted from these samples as per the Trizol RNA extraction protocol and eluted into 8 μl nuclease-free water. RNA was treated with ezDNase (Invitrogen, 11766051), concentration measured using a Nanodrop and then used in Superscript IV First Strand cDNA synthesis (Invitrogen, 18090200) in an RT+ and RT− reaction. Eyes dissected from individuals in the ins17 heterozygous crossings (three different clutches) were treated slightly differently to extract RNA. They were first placed in DNA/RNA shield (Zymogen, R1100), and RNA was extracted from genotyped groups using a column-based RNA extraction kit (Zymogen, R1050) before being used for cDNA synthesis with Superscript IV. RNA amounts used in each reaction were calculated to be consistent between samples. Quantitative PCR was then performed in technical triplicates using the three wild-type sibling and three mutant samples using LightCycler 480 SYBR Green I Master (Roche, 04887352001) with the following primers (note that *pcdh15a/b* primers were designed against the beginning third (at ∼2000 nucleotides) of the mRNA (total 6000-7000 nucleotides in length): *pcdh15a*-F, 5′-TTCGCTCAGGTGTCGTACAG-3′; *pcdh15a*-R, 5′-GACCCCTGGTGCTAAAGTGA-3′; *pcdh15b*-F, 5′-TAATGACAACACGCCAACCTT-3′; *pcdh15b*-R, 5′-GCAGCACTGTGATCACTCCT-3′; Actin-F, 5′-AAGCAGGAGTACGATGAGTC-3′; Actin-R, 5′-TGGAGTCCTCAGATGCATTG-3′.

### TEM

Zebrafish heads were fixed in 2.5% glutaraldehyde in 0.1 M phosphate buffer at 4°C overnight or longer, while the tail was used for genotyping (see ‘CRISPR targeting of *pcdh15b* and genotyping’ section). After genotyping, the heads were sorted and further processed. Samples were washed of fixative using 0.1 M phosphate buffer (3-4×10 min) to ensure no trace of glutaraldehyde and placed in 1% osmium tetroxide for 1 h in the dark. Samples were washed of osmium using 0.1 M phosphate buffer and then dehydrated through an ethanol series in 50% (10 min), 70% (10 min), 80% (15 min), 90% (2×10 min) to 100% (2×10 min) ethanol. Samples were then infiltrated with Spurr's epoxy resin through an ethanol:resin series with increasing concentration of resin (of 3:1 for 30 min, 1:1 for 30 min and 1:3 for 1 h) until they were placed into 100% resin. Resin was allowed to infiltrate the sample tissue overnight, replaced with fresh resin the next day and used for embedding into flat moulds. The resin was allowed to polymerize at 65°C overnight, and samples were stored at room temperature until further processed. Sections were cut using a Leica EM UC6 microtome. One-micrometre semi-thin sections were cut and stained with Toluidine Blue. Semi-thin sections were imaged on a Leica DMI3000 inverted microscope. Then, 100 nm ultrathin sections were cut with a diamond knife and placed on 200-mesh high transmission grid or open-hole slotted grids covered in formvar. Note that slotted grids allowed the whole retina to be visualized and imaged without obstructions. Sections were then stained with 3% uranyl acetate in 50% methanol for 45 min and post-stained with Reynold's lead citrate for 15 min, followed by drying. Samples were imaged using Hitachi HT-7700 at 80 kV and images taken with an attached AMT 11-megapixel digital camera.

### SEM

For analysis of the retina, zebrafish were dark adapted for at least 2 h before fixation to ensure that RPE processes were retracted and would not interfere with OS visualization. In the dark, anesthetized zebrafish were fixed in 4% paraformaldehyde (PFA) at 4°C overnight. The next day, eyes were dissected out and fixed in 2.5% glutaraldehyde in 0.1 M phosphate buffer at 4°C overnight, while the body was used for genotyping. For analysis of the lateral line, fish were placed directly in 2.5% glutaraldehyde overnight at 4°C. Samples were sorted by genotype and then processed further. Samples were washed in 0.1 M phosphate buffer (3×10 min), fixed in 1% osmium tetraoxide for 1.5 h in the dark and then washed in 0.1 M phosphate buffer (3×10 min). Samples were dehydrated in an ethanol series from 50% to 100% (50%, 70% 80%, 90% and two 100% washes for 10 min each) and then placed in an ethanol:hexamethyldisilazane (HMDS) series with increasing concentrations of HMDS (3:1, 1:1, 3:1 washes at 10 min each) until they were in 100% HMDS (washed 2×10 min). HMDS was allowed to evaporate slowly off the samples in the fume hood overnight. The next day, samples were placed on carbon tape on specimen mounts and sputter coated with gold-palladium for visualization in SEM. For examination of the lateral line, samples were mounted directly. However, for photoreceptor examination in the retina, dried samples were carefully fractured with a blunt-ended tool to expose the inner retinal layers and photoreceptor layer, before mounting. Samples were imaged using Hitachi SU3500 at 5 kV with a spot intensity of 30.

### Whole-mount immunofluorescence

Samples (heads for inner ear examination or dissected eyes for photoreceptor examination) were fixed in 4% PFA at 4°C no longer than overnight. Samples were sorted by genotype and the next day washed of fixative using PBST (phosphate buffer saline+0.8% Triton-X). Samples were permeabilized in ice-cold 100% acetone for 10 min at −20°C followed by washes in PBST. Samples were additionally permeabilized with proteinase K (20 µg/ml) in PBST for 1 h at room temperature. Samples were washed in PBST, post-fixed in 4% PFA for 20 min at room temperature and washed. Samples were then blocked in saturation buffer (PBST with 10% goat serum and 1% dimethyl sulfoxide) for at least 1 h at room temperature. Primary antibody/stain diluted in saturation buffer was incubated for 2-3 days at 4°C with agitation. Samples were then washed three times for 1 h each in PBST. If secondary antibody labelling was required, samples were re-blocked in saturation buffer for at least 1 h, and then placed in diluted secondary antibody overnight. The following day, samples were washed in PBST three to four times for 1 h each. Before imaging, samples were infiltrated with 80% glycerol for a couple of hours and placed in glass-bottom imaging dishes for imaging. Images were taken on a Leica TCSSP8 confocal microscope. The following antibodies/stains were used: Alexa Fluor™ 488 Phalloidin (1:100; ThermoFisher Scientific, A12379), Lectin PNA from *Arachis hypogaea* (peanut) Alexa Fluor™ 488 Conjugate (1:100; ThermoFisher Scientific, L21409, RRID: AB_2315178), Wheat Germ Agglutinin Alexa Fluor™ 555 Conjugate (1:100; ThermoFisher Scientific, W32464), mouse anti-acetylated alpha tubulin (1:500; Sigma-Aldrich, clone 6-11B-1, T7451, RRID: AB_609894) and Cy3 goat anti-mouse IgG (1:100; Jackson ImmunoResearch, 111-165-146, RRID: 2491007).

### Immunohistochemistry

Samples were fixed in 4% PFA (in PBS) overnight at 4°C. Samples were cryoprotected by a sucrose series of five successive 30 min washes of increasing sucrose concentration from 5% to 30% sucrose in 1× PBS. Samples were kept overnight in 30% sucrose at 4°C and then exchanged the next day into a 2:1 mixture of 30% sucrose: optimal cutting temperature compound (OCT). Samples were frozen at −20°C until sectioning. Cryosections through the heads and eyes were cut at 20 µm thickness. Immunohistochemistry was performed as previously described (Olsen et al., 2016). The following antibodies/stains were used: anti-Caspase-3 [rabbit, 1:200; cleaved (activated), Asp175, Cell Signaling Technology, 9661, RRID: 2341188], anti-Zpr1 (mouse, 1:250; ZIRC), anti-Zpr3 (mouse, 1:250; ZIRC), anti-rhodopsin (mouse, 1:500; Sigma-Aldrich, clone RET-P1, MAB5316), anti-acetylated alpha tubulin (mouse 1:500; Sigma-Aldrich, clone 6-11B-1, T7451, RRID: AB_609894), Alexa Fluor™ 488 Phalloidin (1:100; ThermoFisher Scientific, A12379), anti-Cacna1fa [rabbit, 1:3000; kindly donated by Michael Taylor (Jia et al., 2014)], anti-Pcdh15 (rabbit, 1:250; NSJ Bioreagents, RQ4652), anti-rabbit Cy3 (1:500; Jackson ImmunoResearch, 111-165 003, RRID: AB_2338000), anti-mouse Cy3 (1:500; Jackson ImmunoResearch, 115-165-146, RRID: 2491007), anti-mouse Cy5 (1:200; Jackson ImmunoResearch, 115-175-166, RRID: AB_2338714) and anti-rabbit Cy5 (1:200; Jackson ImmunoResearch, 111-175-144, AB_2338013). Sections were imaged on a Leica TCSSP8 confocal microscope.

### Western blotting

Eyes were dissected from individuals obtained from Δ7 heterozygous crossings and immediately frozen at −80°C using an ethanol and dry ice mixture and kept at −80°C until genotyping was completed. The eyes were grouped based on genotype (i.e. wild-type, homozygous mutants) and thawed and homogenized in RIPA buffer (Sigma-Aldrich, R0278) containing fresh protease inhibitor (Sigma-Aldrich, P8340) (98 μl RIPA buffer, 2 μl protease inhibitor per 100 μl) using a pestle, followed by homogenization with a fine needle (27 gauge). The sample was incubated in the extraction mixture for at least 30 min at 4°C and then quickly centrifuged to remove debris. Half the sample (equivalent to 14 eyes) was mixed with NuPAGE LDS sample buffer (Invitrogen, NP0007) and 5% β-mercaptoethanol, but not heated prior to being loaded into the gel as this provided better results (Tsuji, 2020). The protein samples were run on a NuPAGE 4-12%, Bis-Tris mini protein gel (Invitrogen, NP0335) at 150 V for 1 h. The protein was transferred to a PVDF membrane at a constant current of 200 mA for ∼3 h or until the largest protein marker (225 kDa) transferred over. The membrane was washed in TBST (20 mM Tris, 150 mM NaCl, 0.05% Tween 20) and then blocked in blocking solution (5% dried skim milk in TBST) at room temperature for 1 h. The anti-Pcdh15 antibody (NSJ Bioreagents, RQ4652) was diluted in blocking solution at 1:1000 and incubated overnight at 4°C. The membrane was washed in TBST and then incubated with the secondary antibody anti-rabbit HRP (Jackson ImmunoResearch, 111-035-045, RRID: AB_2337938) at 1:10,000 in TBST for 30 min at room temperature. The membrane was subsequently washed in TBST. To image, an electrochemiluminescence reaction was performed for 1 min (FroggaBio, Ultrascence Western Substrate, CCH365_Femto) and the membrane was imaged on a ChemiDoc Touch Imaging System (Bio-Rad). The control mouse anti-actin antibody (EMD Millipore, MAB1501, RRID: AB_2223041) used at 1:10,000 was similarly probed and imaged using an anti-mouse HRP secondary antibody (1:10,000; Jackson ImmunoResearch, 115-035-003, RRID: AB_10015289). The control rabbit anti-H2B antibody (ThermoFisher Scientific, PA1-41058, RRID: AB_2118162) used at 1:500 was similarly probed and imaged using an anti-rabbit HRP secondary antibody (1:10,000; Jackson ImmunoResearch, 111-035-045, RRID: AB_2337938).

### Manipulation of light exposure

Prior to 6 dpf, all embryos/larvae were treated similarly. At 6 dpf, light exposure was manipulated. Lux was measured using a digital lux meter (Dr. Meter) at the level of Petri dish placement. In most experiments (excluding the differential light exposure experiments), larvae clutches were moved to the animal facility (kept at 28°C) and were exposed to ∼250 lux light (unmanipulated light in the facility) on a 14 h-10 h light-dark cycle until time of sacrifice.

For the differential light exposure experiments, larvae were assigned to different lighting groups. For the ‘dark’ condition, larvae were covered with a blackout box for 24 h daily. They were only exposed to light during feeding and water changes. For the ‘bright-light’ condition, larvae were placed in the facility under light measuring 500 lux on a 14 h-10 h light-dark cycle. However, in addition to this light, larvae were exposed to daily periods of white light of 3000 lux or 6000 lux intensity for 5 h during the afternoon (12:00-17:00). All groups were exposed to these light treatments from 6 dpf to 10 dpf and then sacrificed.

### Experimental design and statistical analysis

Genotyping was performed on >30 randomly selected larvae to ensure adequate numbers for each experiment. Eyes from at least four individuals or more (see figure legends for more details on number of eyes/animals) were analysed for each group per experimental procedure and time point.

For immunostaining on sections, three consecutive sections in the central retina were examined to ensure consistency across the tissue. For SEM lateral line examination, more than five neuromasts per individual, on average, were used for percentage counts. For SEM retinal examination, at least five eyes (from different individuals) were successfully fractured and examined per genotype. For TEM, retinal sections from the central retina (close to the optic nerve) were used for analysis. When possible, both eyes from an individual were analysed and included in the data. Each dot represents data from one eye (details of number of eyes and individuals examined are in the figure legends). For OS analysis, measurements were taken across the whole retinal section for 4 dpf (Δ7, ins17 and controls), 5 dpf (Δ7 and controls), 10 dpf (Δ7, ins17 and controls) and dark treatment (Δ7 and controls). For 15 dpf (Δ7 and controls), and bright-light treatment (Δ7 and controls), only the central region of the retinal section, delimited by the plexiform layers, was examined for measurements. The numbers of OSs examined for each measurement are included in the figure legends. For CP analysis, only the central retina was used. Included in the CP counts were at least 30 OSs for each eye. Only CPs that were connected to the OS were included in the count. OSs that lacked any CP (i.e. had zero CPs) were not included in the analysis to ensure that we were sampling in a region of the OS in which CPs are consistently observed between genotypes. For synapse analysis, only central retinal synapses were examined (∼20-30 synapses per retinal section per individual eye).

Immunostaining data were processed using ImageJ. Hoechst^+^ cells in the ONL of three consecutive confocal sections (20 µm each) were counted using IMARIS (Bitplane version 7.7.1). The area was measured using ImageJ and averaged for cells/area in the ONL across the three sections. Pyknotic nuclei were counted using ImageJ Cell counter function manually. All TEM measurements were taken on AxioVision SE64 Rel 4.9.1. Statistical analyses were performed using GraphPad Prism 7.0. Statistical tests used included Student's *t*-tests (unpaired, two-tailed) when comparing two groups or one-way ANOVA when comparing three groups. Statistical analyses of the OS morphologies, OS detachment and synapse organization in the different genotypes were performed using Student's *t*-tests (unpaired, two-tailed). Statistical analyses of time-dependent effects of controls and mutants on photoreceptors and the effect of different light treatments on controls and mutants were performed using two-way ANOVA. All error bars represent s.d. Specific details on statistical tests can be found in the figure legends.

## Supplementary Material

Supplementary information
