# Supplementary material for: Usher syndrome type 1-associated gene, pcdh15b, is required for photoreceptor structural integrity in zebrafish
Source: Dis Model Mech. 2021 Dec 7;14(12):dmm048965. doi: 10.1242/dmm.048965 (PMC8669488; doi:10.1242/dmm.048965)
Supplement: Supplementary information [file dmm-14-048965-s1.pdf]

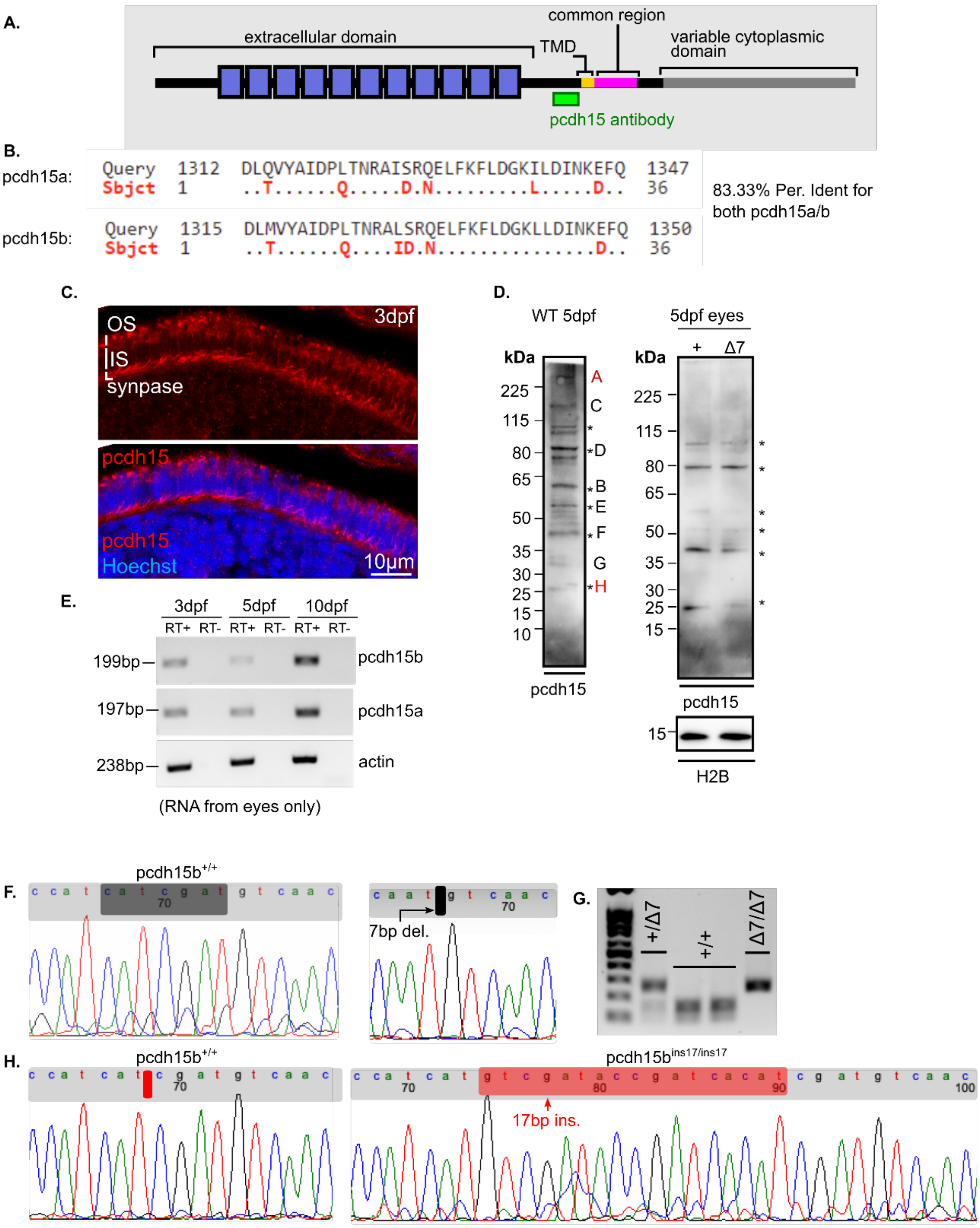

Disease Models & Mechanisms • Supplementary information

Fig. S1. Generation of pcdh15b mutants by CRISPR. (A) Schematic of pcdh15 protein and the relevant domains (TMD= transmembrane domain) and the locations of the pcdh15 antibody. (B) Alignment of the antigen recognized by the pcdh15 antibody and pcdh15a (top) and pcdh15b (bottom). Query= pcdh15a/b protein sequence; Sbjct (red)= Mismatches from the antibody antigen sequence. **(C)** Representative image of pcdh15 expression in the 3dpf retina at the ONL. OS= outer segment, IS= inner segment, ONL= outer nuclear layer, INL= inner nuclear layer. **(D)** Western blot analysis of the pcdh15 antibody in isolated WT and mutant retinas. The * bands are the well-defined bands in each blot. The lettering (A-H) correspond to bands previously seen and described in (Alagramam et al., 2011). **(E)** RT-PCR of *pcdh15a*, *pcdh15b* and control actin mRNA in 3dpf, 5dpf and 10dpf eyes. **(F, H)** Representative sequencing chromatograms of wildtype siblings (left panels) and mutants (Δ7 (F), ins17 (H)) (right panels), with highlighted bands showing the affected regions (*grey* for 7bp deletion and *red* for 17bp insertion). **(G)** In addition to sequencing, the 7bp deletion (but not the 17bp insertion) can be identified by the loss of a ClaI digest site resulting in an uncut large band, as shown in the representative gel.

Disease Models & Mechanisms • Supplementary information

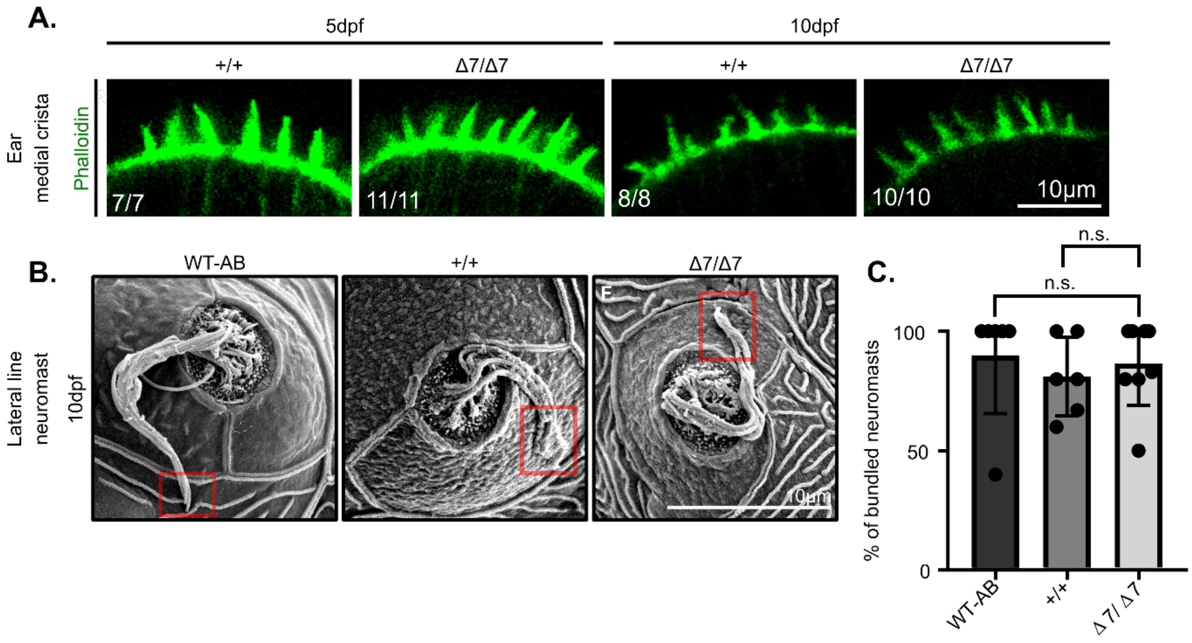

Fig. S2. Hairs cells of the inner ear and lateral line are unaffected in pcdh15b mutants. (A) Representative images of stereocilia, found in hair cells of the medial crista of the inner ear, labelled by phalloidin (*green*) in wildtype siblings (+/+) and pcdh15b mutants (Δ7/Δ7) at 5dpf (n=7, 11) and 10dpf (n= 8, 10). **(B)** Representative SEM images of neuromasts along the lateral line in unrelated wildtype-AB (WT-AB), wildtype siblings (+/+) and pcdh15b mutants (Δ7/Δ7). Red boxes highlight the bundled tips found in each neuromast. **(C)** Quantification of bundled neuromast tips. One-way ANOVA n.s.= p>0.05. Each individual graph point represents the percent of bundled tips from an average of at least 5 neuromasts examined per individual (WT-AB n=6, +/+ n=6, Δ7/Δ7 n=8).

Disease Models & Mechanisms • Supplementary information

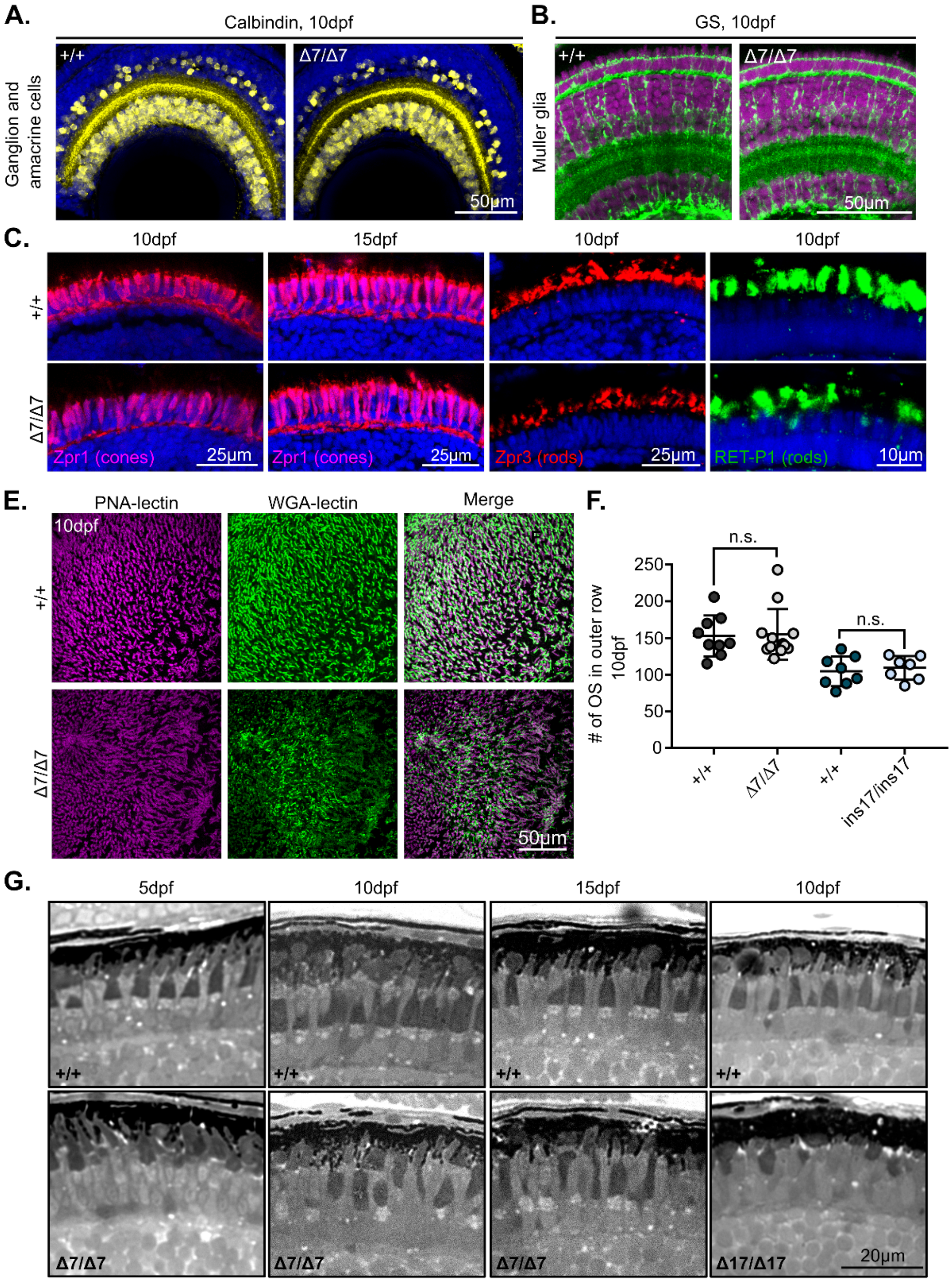

Fig. S3. Abnormalities in the pcdh15 mutant retina is restricted to the cone and rod photoreceptors. (A) Representative images of calbindin (*yellow*), a ganglion and amacrine cell marker, and **(B)** glutamine synthetase (GS) (*green*), a Muller glia cell marker, at 10dpf in wildtype sibling and Δ7

Disease Models & Mechanisms • Supplementary information

mutant retinas. Nuclei are counterstained with Hoechst (*Blue (A) /magenta (B)*). **(C)** Representative images from different cone and rod photoreceptor markers in wildtype siblings and Δ7 mutants at 10dpf and 15dpf. Zpr1 labels red-green double cone cell bodies (*magenta*). Zpr3 labels a rod outer segment component (*red*). RET-P1, labels rhodopsin in outer segments (*green*). Nuclei are counterstained with Hoechst (*blue*). **(E)** Whole mount immunostaining of the retina, in wildtype siblings (n=17) and Δ7 mutants (n=10) by WGA-lectin (*green*), and PNA-lectin (*magenta*) at 10dpf. **(F)** The number of OS found in the 'outer row' of the photoreceptor layer of wildtype siblings and pcdh15b mutants at 10dpf measured in TEM images across the entire ONL. **(G)** Representative semi-thin sections of the photoreceptor layer of pcdh15b mutants (Δ7, ins17) and their wildtype siblings from 5-15dpf.

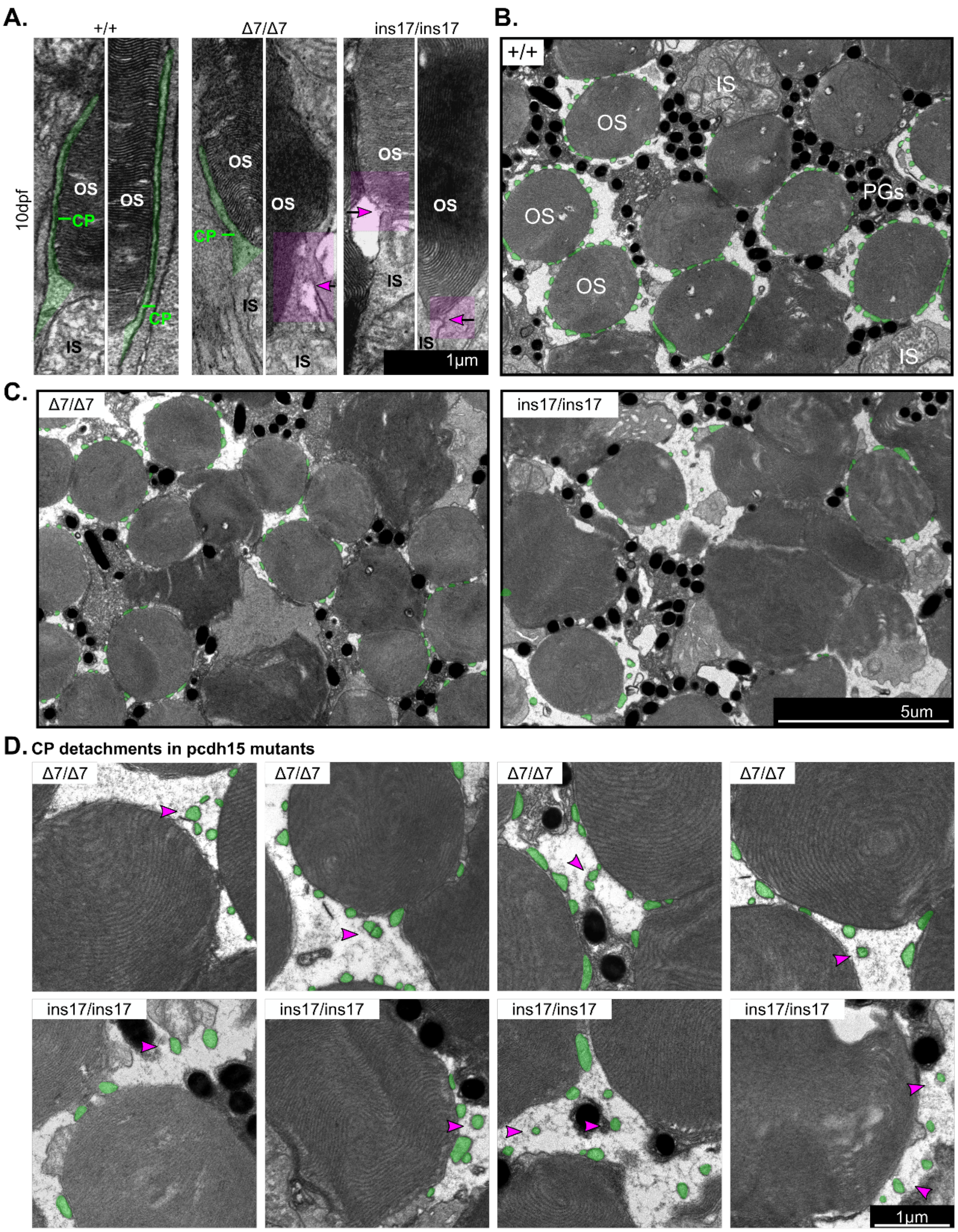

Fig. S4. CPs become detached and are eventually lost/reduced in pcdh15b mutant photoreceptors. (A) Representative TEM images of CPs found on the side of OS in wildtype sibling and pcdh15b mutant photoreceptors. CPs are highlighted in green. Regions in pcdh15b

Disease Models & Mechanisms • Supplementary information

mutants that have lost CPs are highlighted in magenta and shown with a magenta arrow. **(B-C)** Overview representative images of horizontal TEM sections through the photoreceptor layer in wildtype siblings (B) and mutants (C). CPs surrounding photoreceptor OS are coloured green. Abnormal OS shape in mutants can also be seen in these sections. **(D)** Multiple examples from horizontal TEM sections showing CP detachment (magenta arrowhead) in the different pcdh15b mutants (Δ7 and ins17). All visible CPs are coloured green. CPs= calyceal processes, OS= outer segment, IS= inner segment, PGs= pigment granules.

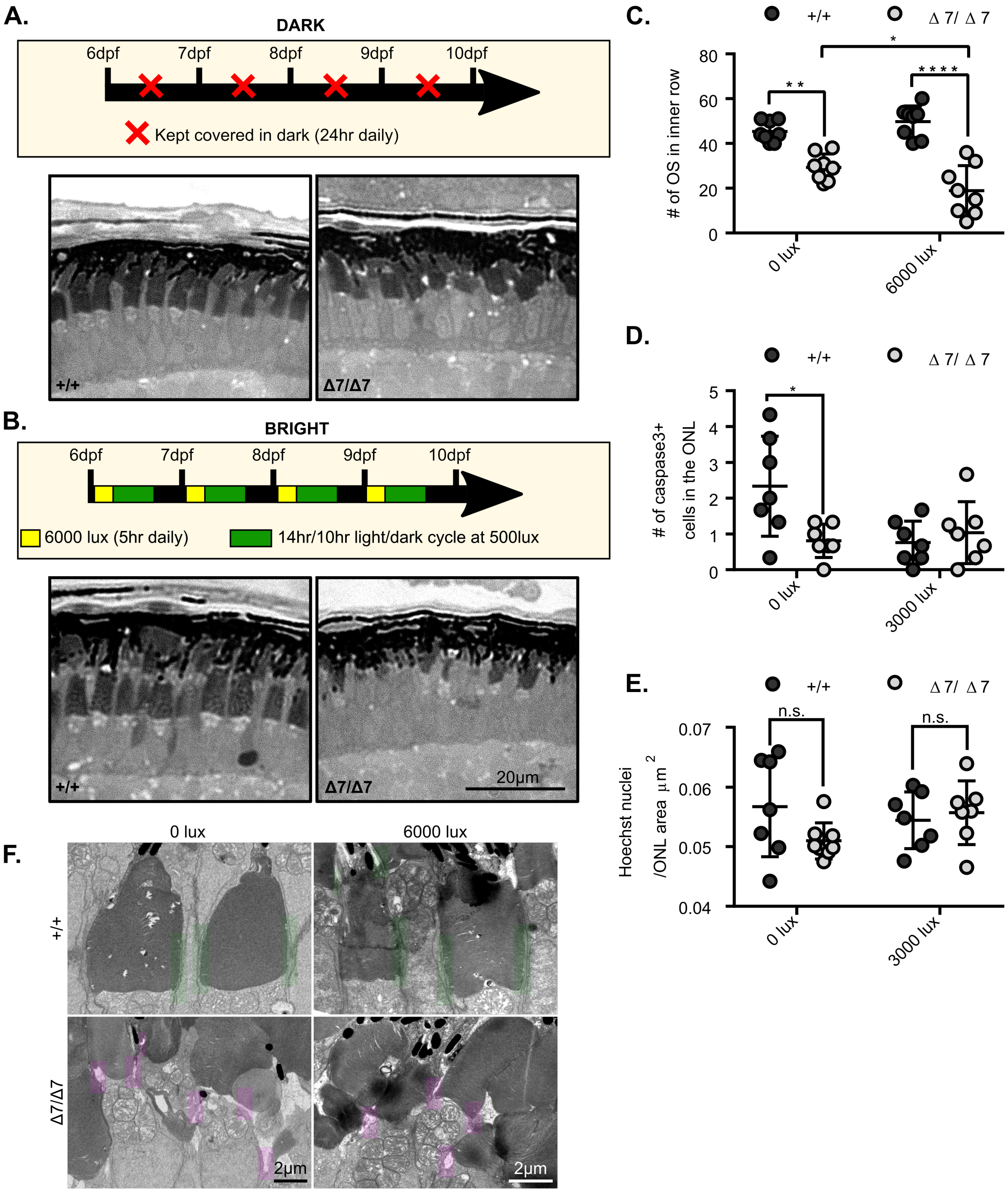

Fig. S5. Light exposure influences the severity of abnormalities in the photoreceptors of *pcdh15b* mutants. (A-B) Wildtype siblings and Δ7 mutants were grown under dark conditions (0 lux) (A) or bright light conditions (6000 lux) (B) from 6-10dpf. Representative semi-thin retinal sections (1μm thick) of

Disease Models & Mechanisms • Supplementary information

wildtype sibling (n=8 eyes (0 lux), 8 eyes (6000 lux)) and Δ7 mutants (n=8 eyes (0 lux), 8 eyes (6000 lux)) under each condition at 10dpf. **(C)** Quantification of the number of OS in the inner row across a semi-thin section in the different light conditions. **(D-E)** Quantification of the number of caspase-3+ cells in the ONL/section (D) and photoreceptor density (Hoechst nuclei/ONL area) between wildtype siblings (n=7 eyes (0 lux), 7 eyes (3000 lux)) and Δ7 mutants (n=7 eyes (0 lux), 7 eyes (3000 lux)) (E) under the different light conditions. **(F)** Example images of CPs visualized by TEM in dark and bright conditions in wildtype siblings and mutants. Green boxes highlight attached CPs, magenta boxes highlight detached CPs. Statistical tests in (C, D, E) were performed using two-way ANOVA. n.s., non-significant, *P=≤0.05, **P=≤0.01, ****P=≤0.0001.

Disease Models & Mechanisms • Supplementary information

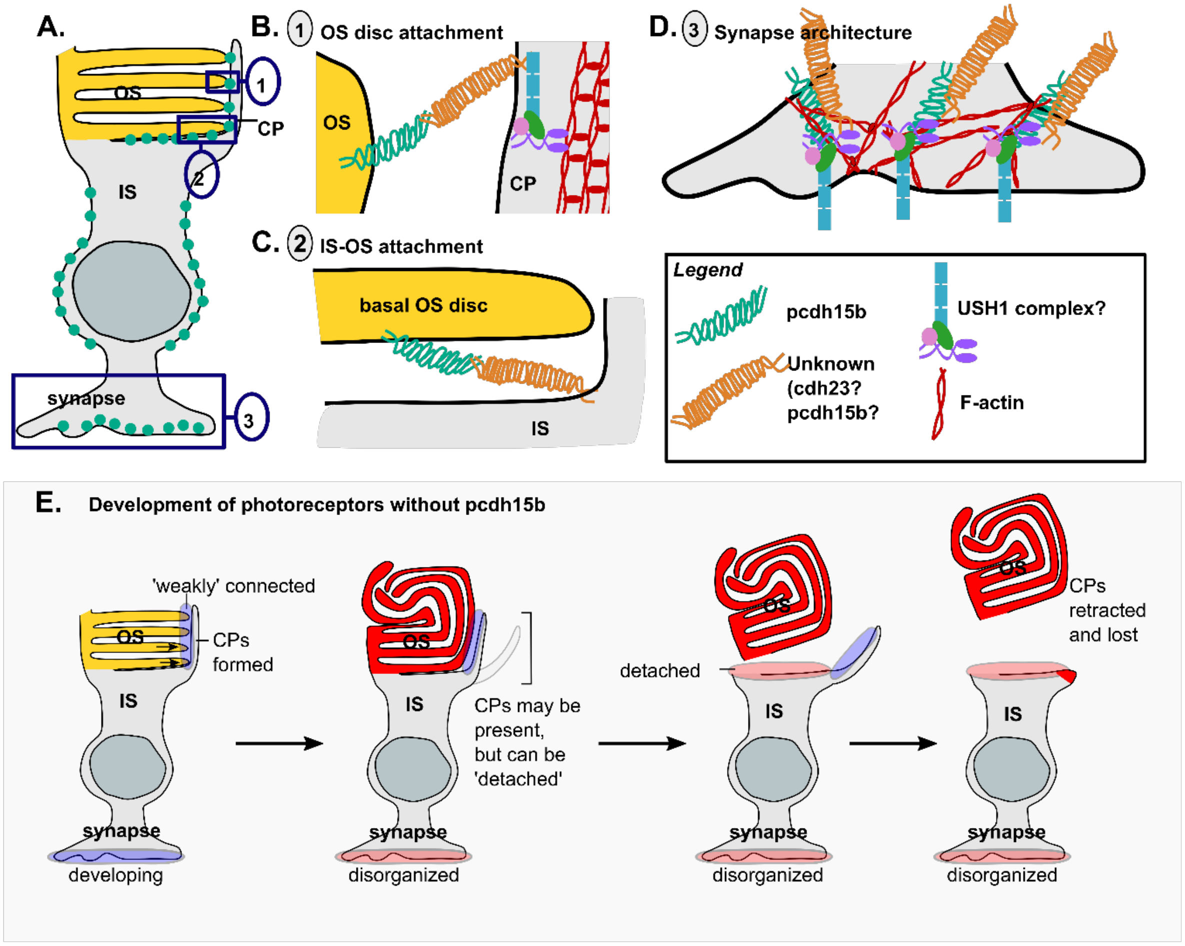

Fig. S6. Model of pcdh15b function in the zebrafish retina. These data suggest that zebrafish pcdh15b functions at 3 main subcellular compartments in the photoreceptor: the CPs, the IS-OS junction and the synapse. **(A)** Schematic of the photoreceptor and location of pcdh15b expression based on our pcdh15 expression data. OS= outer segment, IS= inner segment, CP= calyceal processes. Green dots are locations of pcdh15 expression. Blue boxes are the regions examined more closely in (B-D). **(B)** Close up OS disc schematic of the attachment between OS discs and the CP mediated by pcdh15, and possible other proteins not yet clarified in zebrafish. **(C)** Close up of the IS-OS attachment between the basal OS disc and the IS plasma membrane by pcdh15 **(D)** Close up schematic of how pcdh15 (and other USH1 proteins) organized the actin

Disease Models & Mechanisms • Supplementary information

cytoskeleton and synapse in photoreceptors. (E) Schematic of the photoreceptor developing in the absence of pcdh15b. Images left to right: (First) As the photoreceptor develops the discs of the OS start to move towards a formed CP protrusion, however it does not form a strong attachment. (2) Without a strong attachment, the OS discs instead continue to grow in abnormal directions. By the time the synapses mature, they are disorganized. (3) The OS will eventually detach as pcdh15 is not there to secure the attachment to the IS plasma membrane. CPs will start to fall away. (4) The detached OS will float further away from the rest of the photoreceptor and eventually the CP will be completely lost and retracted. Retraction may occur earlier in the previous stages. Red colouring in the image highlights regions that have become defective in the photoreceptor.

Disease Models & Mechanisms • Supplementary information
